# CCDC22 and CCDC93, two potential retriever-interacting proteins, are required for root and root hair growth in Arabidopsis

**DOI:** 10.3389/fpls.2022.1051503

**Published:** 2022-12-22

**Authors:** Connor D. Lewis, Jill C. Preston, Mary L. Tierney

**Affiliations:** Department of Plant Biology, University of Vermont, Burlington, VT, United States

**Keywords:** retriever1, CCDC222, CCDC933, endosomal trafficking4, VTI135, root and root hair7, Arabidopsis, retromer

## Abstract

Endomembrane trafficking is essential for plant growth and often depends on a balance between secretory and endocytic pathways. VPS26C is a component of the retriever complex which has been shown to function in the recycling of integral plasma membrane proteins in human cell culture and is part of a core retriever complex in Arabidopsis that is required for root hair growth. In this work, we report a characterization of the Arabidopsis homologues of CCDC22 and CCDC93, two additional proteins required for retriever function in humans. Phylogenetic analysis indicates that *CCDC22* (AT1G55830) and *CCDC93* (AT4G32560) are single copy genes in plants that are present across the angiosperms, but like *VPS26C*, are absent from the grasses. Both *CCDC22* and *CCDC93* are required for root and root hair growth in Arabidopsis and localize primarily to the cytoplasm in root epidermal cells. Previous work has demonstrated a genetic interaction between VPS26C function and a VTI13-dependent trafficking pathway to the vacuole. To further test this model, we characterized the *vti13 ccdc93* double mutant and show that like *vps26c*, *ccdc93* is a suppressor of the *vti13* root hair phenotype. Together this work identifies two new proteins essential for root and root hair growth in plants and demonstrate that the endosomal pathway(s) in which CCDC93 functions is genetically linked to a VTI13-dependent trafficking pathway to the vacuole.

## 1 Introduction

Endosomal trafficking is essential for almost all aspects of plant growth. Movement of cargo throughout the endomembrane system requires the function of SNAREs (Soluble N-ethylmaleimide-sensitive factor Attachment protein Receptors), membrane proteins that mediate vesicle fusion between two intracellular compartments and RAB GTPases that regulate these processes (for review, Ito and Uemura, 2022). In plants, many of these proteins are encoded by gene families. The VTI family of SNAREs exhibits a conserved function in yeast and plants by trafficking cargo to either the lytic or storage vacuole (Surpin et al., 2003). However genetic analysis in Arabidopsis indicates that each VTI SNARE contributes uniquely to plant growth by controlling specific developmental processes. While VTI11 is essential for shoot gravitropism (Kato et al., 2002), VTI12 functions during autophagy (Surpin et al., 2003) and VTI13 is essential for root hair growth and cell wall organization (Larson et al., 2014). These differences in the contribution of each VTI gene family member may be the result of their cell type expression during development, different protein/protein interactions necessary for their function, or differences in the cargo associated with each of the VTI-dependent trafficking pathways.

While trafficking proteins to the lytic vacuole for degradation is important for cellular homeostasis, endosomal trafficking pathways between the *trans*-Golgi network (TGN) and the lytic vacuole (in plants) or the lysosome (in animals) also include the retromer. The retromer was first described in yeast where it was shown to consist of a large subunit containing VPS26, VPS29 and VPS35 and a small subunit consisting of two sorting nexins (Seaman et al., 1998). Homologues of each of these proteins have been identified in most eukaryotic organisms and their roles in trafficking pathways important for growth and development, response to the environment, and disease have been widely studied. However, unlike yeast, genetic studies indicate that the large and small subunits of the retromer may function separately in both plant and animal systems (Pourcher et al., 2010; Zelazny et al., 2013; Kvainickas et al., 2017; Simonetti et al., 2017).

In *Arabidopsis thaliana* (Arabidopsis) the large retromer subunit is encoded by VPS35, VPS29 and VPS26. VPS35 and VPS26 are each encoded by three gene family members while VPS29 is a single copy gene. Arabidopsis also encodes six sorting nexins, SNX1, SNX2a and SNX2b, SNX 3, SNX4 and SNX5 that dimerize as pairs to form the small subunit of the retromer. Genetic analysis of these proteins has shown that *vps35a vps35b vps35c*, *vps35b vpas35c*, *vps26a vps26b* and *vps29* have severe growth phenotypes (Jaillais et al., 2007; Yamazaki et al., 2008; Zelazny et al., 2013) while the growth patterns of *vps35* and *vps26* single mutants are largely unaffected. This suggests that while retromer function is necessary for many aspects of growth, the VPS35a, b, and c isoforms and VPS26a and b isoforms may be able to compensate for each other in *vsp35* and *vps26* single mutants. In addition, genetic analysis has identified several aspects of plant growth in which specific conformations of the retromer is required including auxin signaling (Kleine-Vehn et al., 2008), cell polarity (Jaillais et al., 2007), plant defense (Munch et al., 2015) and shoot gravitropism (Hashiguchi et al., 2010).

We recently characterized a member of the VPS26 gene family in Arabidopsis (VPS26C; AT1G48550) and showed that it is required for root hair growth and is a genetic suppressor of the *vti13* root and root hair phenotypes (Jha et al., 2018). VPS26C shares only 28% amino acid sequence identity with VPS26A and VPS26B in Arabidopsis but shares greater than 50% amino acid sequence identity with a subset of VPS26C homologues found in both animals and plants. Phylogenetic analysis showed that VPS26C is part of an ancient clade (Koumandou et al., 2011), suggesting that it may have evolved a novel retromer function. Phylogenetic analysis of VPS26C homologues in plants showed that VPS26C is present in most angiosperms with the notable exception of the grasses (Jha et al., 2018).

The function of the VPS26C complex in human cell culture has been linked to the recycling of plasma membrane protein cargo that is distinct from those proteins recycled by the retromer (McNally et al., 2017). Based on this newly described function, McNally et al. named the VPS26C complex “Retriever”. Using a proteomic approach, McNally et al., 2017 identified a number of other proteins that complex with retriever that are essential for the recycling of plasma membrane proteins. Two of these, CCDC22 (AT1G55830) and CCDC93 (AT4G32560) have orthologs in Arabidopsis. In this paper we characterize the role(s) of these proteins in seedlings and show that they are essential for both root and root hair growth. Phylogenetic analysis of CCDC22 and CCDC93 in angiosperms demonstrates that they share an evolutionary history with VPS26C. Lastly, suppressor analysis of the *vti13* phenotype in the *vti13 ccdc93* double mutant also provided genetic evidence that like VPS26C, CCDC93 shares a genetic interaction with a VTI13-dependent trafficking pathway to the vacuole that is required for root hair growth in plants.

## 2 Materials and methods

### 2.1 Plant materials, T-DNA insertion verification, and growth conditions

#### 2.1.1 Plant materials

Arabidopsis (Colombia-0) was used for all experiments involving wild type and mutant analysis. SALK lines used for these studies were obtained from the Arabidopsis Biological Research Center (ARBC) and include: SALK_047800 (*ccdc22-1*), SALK_039808C (*ccdc93-*1), SALK_142324C (*ccdc93-2*) and SALK_075261 (*vti13*). To generate the *vti13ccdc93* double mutant *vti13* was crossed to *ccdc93-1*, the resulting F1 progeny were selfed and double mutants were identified in the F2 generation. Seed was collected from homozygous *vti13 ccdc93* F2 individuals and F3 seedlings were used to analyze root hair length. PCR was used to confirm the homozygosity of all SALK mutants analyzed in this study by utilizing gene specific primers and the *Agrobacterium tumefaciens* T-DNA left border-specific primer LB1.3 (**Figures S7**, **S9**, **S10**). See **Table S1** for primer sequences used for genotyping.

#### 2.1.2 Growth conditions

Plants grown to maturity were sown in Metro-Mix 360 professional growing mix (Sungro Horticulture, Agawam, MA, USA), and grown in growth chambers (Conviron, Pembina, ND, USA) at 20°C under conditions of continuous light (43umol/m^2^/s). Seedlings were grown at room temperature under continuous cool white light (53 umol/m^2^/s) on 1X MS plates [1.3% agarose (Invitrogen #16500500), 1X Murashige and Skoog (MS) salts (Sigma-Aldrich #M5524), 1% (w/v) sucrose, 1X Gamborg’s vitamin solution (Sigma-Aldrich #G1019), and 5 mM 4-morpholineethanesulfonic acid hydrate (MES), pH 6 (Sigma-Aldrich #M8250)].

### 2.2 Phylogenetic analysis

Arabidopsis CCDC22 (AT1G55830) and CCDC93 (AT4G32560) protein sequences were used to conduct plant and animal-specific protein-protein BLAST searches in NCBI (https://blast.ncbi.nlm.nih.gov/Blast.cgi) and Phytozome (https://phytozome-next.jgi.doe.gov/). To confirm the loss of CCDC22 and CCD93 from grasses, BLAST queries were made specifically for Poaceae in NCBI, or rice (*Oryza sativa*), maize (*Zea mays*), *Brachypodium distachyon*, and *Setaria viridis* in Phytozome. Protein sequences representative of land plants and animals were initially aligned with the program MAFFT v7.215 (https://www.ebi.ac.uk/Tools/msa/mafft/) (Katoh and Standley, 2013), with follow-up manual alignment by eye in Mesquite v3.2. Regions of each protein that were highly divergent across taxa were trimmed out of each alignment, and phylogenetic analyses were conducted using 423 and 270 amino acid characters for AtCCDC22 and AtCCDC93, respectively. Bayesian phylogenetic analyses were conducted in MrBayes v3.2.7a (Ronquist et al., 2012) using default parameters for protein sequences on the CIPRES Science Gateway v3.3 and setting the runs to 10 million generations. Majority-rule consensus trees were visualized in FigTree v1.4.4 (Rambaut, 2009), with animal sequences (amino acid alignments) or bryophytes (nucleotide alignments) used as outgroups. To confirm topologies among plant genes, the same analyses were conducted with aligned nucleotide sequences based on CDS regions.

### 2.3 Analysis of *ccdc22* and *ccdc93* seedling phenotypes

Arabidopsis seeds were sterilized in a 20% (v/v) bleach solution with shaking at room temperature. They were then washed 6X in 1 ml of sterile distilled water followed by centrifugation. Sterilized seeds were stored in sterile water in the dark at 4°C for at least 12 hours before being plated on 1X MS media containing 1.3% (w/v) agarose. The petri plates were sealed with micropore tape, positioned vertically, and grown as described above. To analyze root and root hair lengths, 5-day-old seedlings were imaged *in situ* on plates using a Leica MZ 8 Stereomicroscope equipped with a Spot Insight 2 camera. Roots were imaged at 6.3X and root hairs at 50X. Seedlings whose roots grew straight into the media were not included in our analyses as only seedlings with roots that grew across the surface of the media were imaged. Root hair images were taken such that the first initiating root hairs observed at the tip of the root were positioned to the bottom of the frame and the root hairs were measured, starting at the top of the frame (1.7mm from the first initiating root hair). Fifteen root hairs per seedling were measured and 10 seedlings per genotype were analyzed for 3 independent biological replicates. For root measurements at least 22 roots per genotype for each of 3 biological replicates were measured. Complementation of these phenotypes was confirmed in 3 independent transgenic lines expressing either the CCDC22-RFP or CCDC93-RFP constructs. ImageJ was used to measure root and root hair lengths of all genotypes studied. Results presented represent the average of the three independent biological replicates. Statistical analyses (ANOVA and Tukey’s Honestly Significant Difference Test) were performed using the R package agricolae (de Mendiburu, 2021).

### 2.4 Plasmid construction and transgenic plant generation

#### 2.4.1 CCDC22:CCDC22-RFP construct

Initially, a *CCDC22* genomic fragment containing the coding sequence was cloned into pENTR/D-TOPO (ThermoFischer #K240020) after being amplified from wild type DNA using the primers ccdc22_pENTR_F and ccdc22_R_noSTOP (**Table S1**). The *CCDC22* promoter (1,897 base pairs (bp) directly upstream of the *CCDC22* initiating ATG) was also cloned into pENTR/D-TOPO after being amplified from wild type DNA using the primers 22proSacIpENTR_F and 22proSpeIpENTR_R (**Table S1**). Q5 site-directed mutagenesis (New England Biolabs #E0554) was performed following the manufacturer’s directions and using the primers 22proMut_F and 22promutR, which resulted in the removal of an internal SpeI site from the *CCDC22* promoter. The mutagenized plasmid containing the CCDC22 promoter sequence was digested with SpeI (NEB #R0133) and SacI (NEB #R0156) and the insert was gel purified in preparation for downstream cloning steps. pB7RWG2.0 (Karimi et al., 2002) was also digested with SpeI and SacI to remove the 35S promoter and generate compatible sticky ends for ligation. Following gel purification, the promoterless pB7RWG2.0 vector was used in a ligation reaction with the purified *CCDC22* endogenous promoter fragment. The plasmid resulting from this ligation was used in a LR clonase II reaction (Thermofisher #11791020) with the pENTR/D-TOPO plasmid containing the *CCDC22* genomic coding sequence (without a stop codon) to generate the final *CCDC22:CCDC22-RFP* construct.

#### 2.4.2 CCDC93:CCDC93-RFP construct

Initially, the *CCDC93* genomic sequence without its stop codon was amplified using ccdc93_pENTR_F and ccdc93_R_noSTOP. The 1,829 bp of DNA directly upstream of the *CCDC93* translational start codon were cloned into pENTR/D-TOPO after being amplified from wild type genomic DNA with the primers 93proSacIpENTR_F and 93proSpeIpENTR_R (**Table S1**). Using Q5 site directed mutagenesis with the primers 93proMut_F and 93proMut_R, an internal SpeI site was removed from the *CCDC93* promoter. Digestion of the mutagenized plasmid with SpeI and SacI released the *CCDC93* promoter, allowing for its purification and ligation into the SpeI and SacI digested, promoterless pB7RWG2.0 vector (Karimi et al., 2002). This plasmid was used in LR clonase II reactions with the pENTR/D-TOPO plasmid containing the *CCDC93* genomic coding sequence (without a stop codon) to generate the final construct.

#### 2.4.3 Plant transformations

The final *CCDC22:CCDC22-RFP*, and *CCDC93:CCDC9-RFP* constructs were transformed into *Agrobacterium tumefaciens* GV3101 prior to triple selection on Luria Broth plates supplemented with rifampicin [10mg/mL], gentamicin [25mg/mL], and spectinomycin [150mg/mL]. *CCDC22* and *CCDC93* RFP fusion constructs were transformed into their respective homozygous mutant backgrounds *via* the floral dip method to select for transgenic plants (Clough and Bent, 1998). T0 seed was harvested, germinated and T1 plants were selected for BASTA resistance. Seed collected from individual T1 plants was germinated and individual T2 plants that displayed resistance to BASTA were used to identify three independent homozygous T3 lines. Three independent T3 lines were assayed for complementation of the short root and root hair phenotypes of the *ccdc22-1* and *ccdc93-1* mutants and the transgenic lines with the strongest fluorescence under the confocal were utilized in future analyses.

### 2.5 Identification of the splice model for CCDC22 and CCDC93 in Arabidopsis

To determine the splicing model used to generate *CCDC22* and *CCDC93* transcripts *in vivo*, we synthesized full length cDNAs for both *CCDC22* and *CCDC93*. Qiagen’s RNeasy plant mini kit was used to isolate RNA from 100 mg of 5-day-old whole seedling tissue grown on 1X MS plates. First strand cDNA was synthesized using BioRad’s iScript cDNA synthesis kit according to manufacturer’s instructions. This first-strand cDNA was used in PCR reactions with either CCDC22_pENTR_F and CCDC22_R_Stop primers or with CCDC93_pENTER_F and CCDC93_R_Stop primers (see **Table S1** for primer sequences) to amplify the *CCDC22* or *CCDC93* transcripts, respectively. The CCDC22 and CCDC92 cDNAs were sequenced and used to predict the amino acid sequence of both proteins.

### 2.6 Relative gene expression

RNA was isolated from seedlings grown on 1X MS media for five or seven days using a Qiagen RNeasy Plant Mini kit (Qiagen #74904). The total RNA concentrations were measured using a Nanodrop (ThermoFisher) and 1mg of total RNA was used to produce first strand cDNA using iScript cDNA synthesis reactions (Bio-Rad #1708891). The cDNA was diluted 1:10 and used with iTaq Universal SYBR Green Supermix (Bio-Rad #1725121) and a StepOnePlus Realtime PCR System (Applied Biosystems) to generate primer efficiencies, cycle threshold values, and melt curves for relative gene expression experiments. Three biological and three technical replicates were used per experiment. Differential expression levels of target genes were normalized to *ACT2* (AT3G18780) expression levels. Two tailed Student’s t-tests were performed in Microsoft Excel to determine if statistically significant differences in the levels of expression of target genes were observed. The primer sequences used are indicated in **Table S1**.

### 2.7 Confocal analysis

Transgenic seedlings expressing *CCDC22:CCDC22-RFP* or *CCDC93:CCDC93-RFP* were grown on 1X MS media for 5 days and the intracellular localization patterns of these fusion proteins in root epidermal cells were analyzed. Three independent transgenic lines were investigated per construct. Confocal microscopy analysis was performed using either a Dragonfly 200 spinning disc confocal microscope (Andor Technology, Inc) on a Leica DM6 B base and equipped with a Zyla 4.2 Mpixel sCMOS camera or using a Nikon A1R-ER point scanning confocal microscope supported by NIH award number 1S10OD025030-01 from the Office of Research Infrastructure Programs. RFP fluorescence was visualized by excitation at 561 nm and a 595/50 nm emission filter. Post-acquisition modifications were made in FIJI and Adobe photoshop (Schindelin et al., 2012).

## 3 Results

### 3.1 ATCCDC22 and ATCCDC93 share significant amino acid sequence homology with their human homologues

We have previously characterized a retriever-like complex in Arabidopsis composed of VPS35A, VPS29 and VPS26C that is required for root hair growth and genetically interacts with a VTI13-dependent trafficking pathway to the lytic vacuole (Jha et al., 2018). This core retriever complex is conserved in many eukaryotes and has been shown to be involved in the recycling of plasma membrane proteins in human cell culture (McNally et al., 2017). These studies identified several additional proteins that are necessary for retriever function. Two of these proteins, CCDC22 and CCDC93, have single copy homologues in Arabidopsis. Expression of both genes appear similar during growth and can be detected in most plant tissues (eFP Browser at br.utoronto.ca). In addition, both *CCDC22* and *CCDC93* are expressed at a lower level in *vps26c* seedlings when compared with wild type seedlings, suggesting that expression of these genes and the core retriever may be co-regulated (**Figure S1**). These results support a model in which CCDC22 and CCDC93 may function in a common pathway with the VPS26C retriever complex in controlling root hair growth.

**Figure 1:**
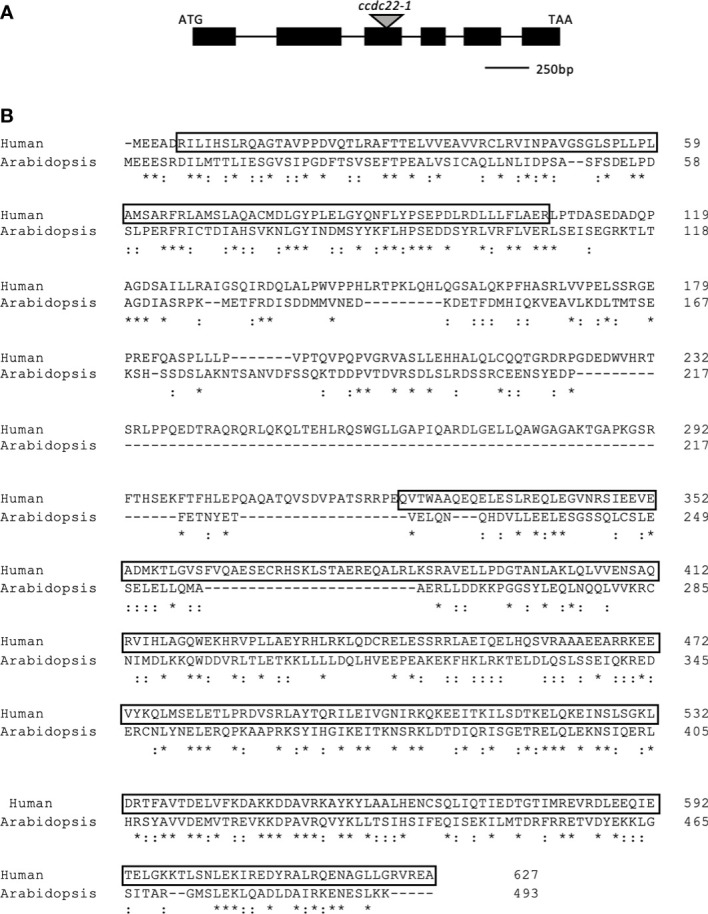
Gene model for *Arabidopsis thaliana CCDC22.* **(A)** Gene model for *ATCCDC22* as determined by aligning the *ATCCDC22* genomic sequence with sequenced, full length *CCDC22* cDNA. The black boxes represent exons and the lines introns. The gray triangle indicates the location of the indicated T-DNA insertion. Image is drawn to scale. **(B)** Human and Arabidopsis CCDC22 Protein Alignments. Alignment of *in silico* translated *ATCCDC22* transcript and the amino acid sequence of HSCCDC22. There is 49% amino acid sequence similarity between the two sequences. The boxed sequences indicate the residues of the N-terminal NN-CH domain and the C-terminal coiled-coil domain. *represents identical amino acids,: represents similar amino acids.

Comparison of the amino acid sequence of the Arabidopsis and human CCDC22 proteins shows that they share 50% amino acid sequence similarity (**Figure 1**). A major distinction between CCDC22 in Arabidopsis and humans is the presence of an internal deletion within the Arabidopsis coding sequence. The predicted amino acid sequence for ATCCDC22 was determined by sequencing a full-length cDNA (**Figure S2**); comparison of this sequence with the three predicted splicing models for *ATCCDC22* confirmed that splicing model two (TAIR website) is utilized *in vivo*. HSCCDC22 is bimodular with a NDC80/NUF2-calponin homology (NN-CH) domain at its N-terminus (amino acids 6-107) and a coiled-coil domain at its C-terminus (amino acids 323-627) (Schou et al., 2014). When comparing the human and Arabidopsis proteins, the NN-CH domain represents one of the most highly conserved stretches of amino acids within the coding sequence, with 59.8% amino acid similarity.

**Figure 2:**
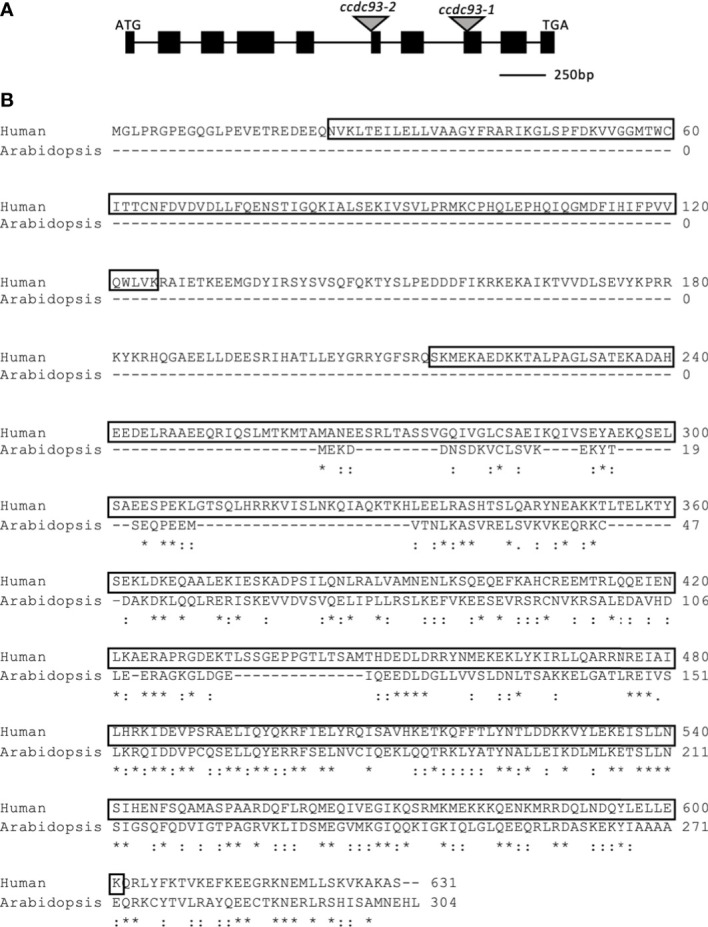
Gene model for *Arabidopsis thaliana CCDC93*. **(A)** Gene model for *ATCCDC93* as determined by aligning the *ATCCDC93* genomic sequence with sequenced, full length *CCDC93* cDNA. The black boxes correspond to exons and the lines introns. The gray triangles show the locations of the integration events of the indicated T-DNA insertion lines. Image is drawn to scale. **(B)** Human and Arabidopsis CCDC93 Protein Alignments. *ATCCDC93* cDNA sequence was translated *in silico* and aligned to HSCCDC93 amino acid sequence. The boxed amino acids correspond to the N-terminal NN-CH domain and the C-terminal coiled-coil domain. ATCCDC93 displays 55% amino acid similarity with the C-terminal half of HSCCDC93. *represents identical amino acids,: represents similar amino acids.

The ATCCDC93 amino acid sequence is truncated with respect to the human CCDC93 homologue and only has amino acid sequence homology to the C-terminal half of the human protein. However, within this region the Arabidopsis and human proteins exhibit 58% amino acid similarity (**Figure 2**). The predicted amino acid sequence for ATCCDC93 was determined by sequencing a full-length cDNA (**Figure S3**) and comparison of this sequence to the predicted splicing models confirmed that splicing model three (TAIR website) is utilized *in vivo*. HSCCDC93 is predicted to have a similar structure as HSCCDC22, as they both have N-terminal NN-CH domains and C-terminal coiled coil domains. However, unlike ATCCDC22, ATCCDC93 has completely lost the NN-CH domain.

### 3.2 CCDC22 and CCDC93 are highly conserved proteins that have been lost in the grass family

Phylogenetic analysis of VPS26 genes in eukaryotes has shown that VPS26C sequences form a distinct clade from those of VPS26A and VPS26B and provides evidence for an ancient duplication within the VPS26 gene family prior to the diversification of plants and animals (Koumandou et al., 2011). Furthermore, phylogenetic analysis of VPS26C sequences in plants revealed that VPS26C has been lost from the grass family (Poaceae) (Jha et al., 2018).

**Figure 3:**
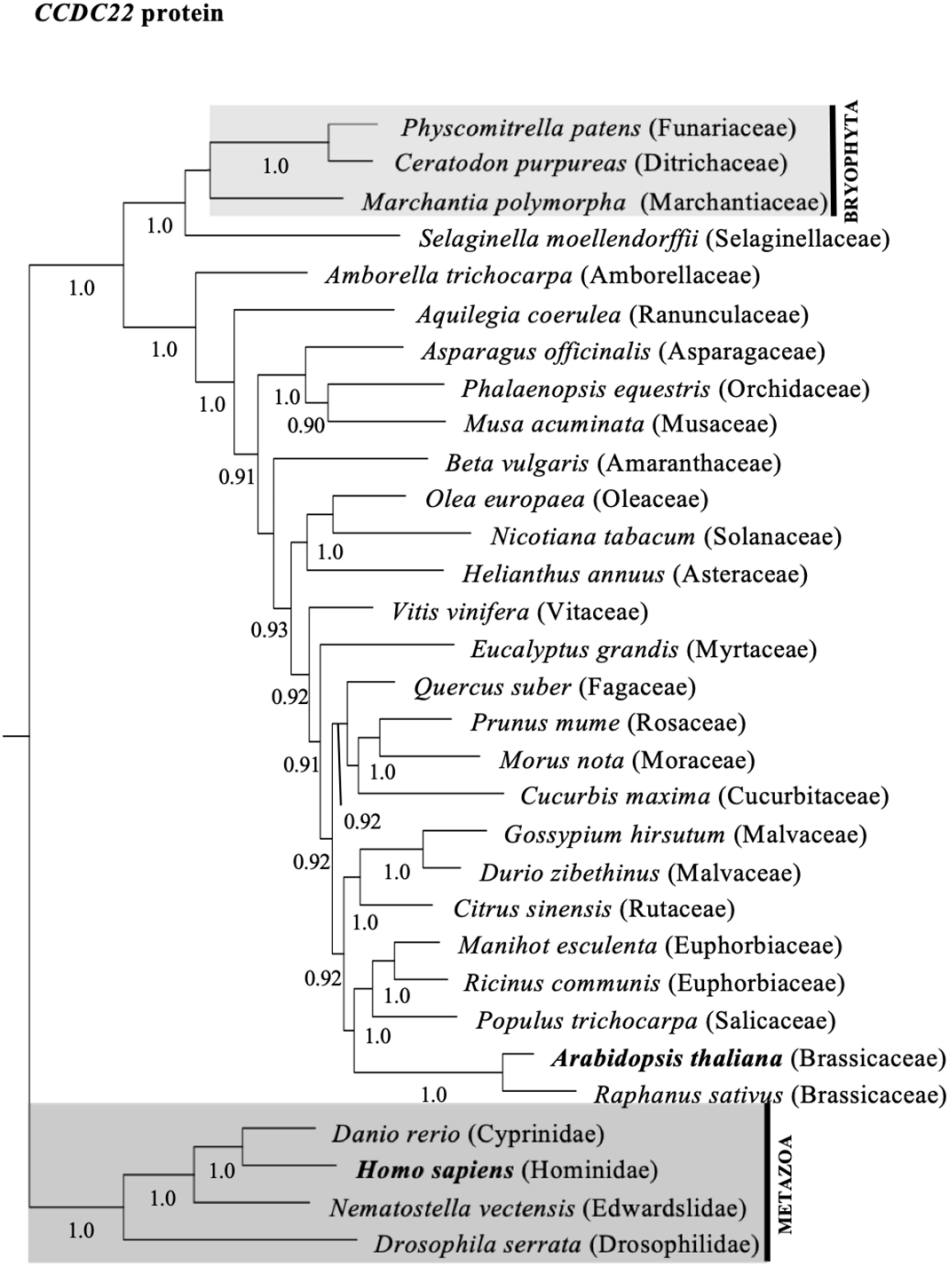
Majority rule consensus tree based on Bayesian phylogenetic analyses of aligned CCDC22 protein sequences. Numbers below branches denote Bayesian posterior probabilities above 0.89. CCDC proteins/genes from Arabidopsis and humans are in bold, and shaded boxes highlight animals (metazoans; dark grey box) and bryophytes (bryophyta; light grey box).

**Figure 4:**
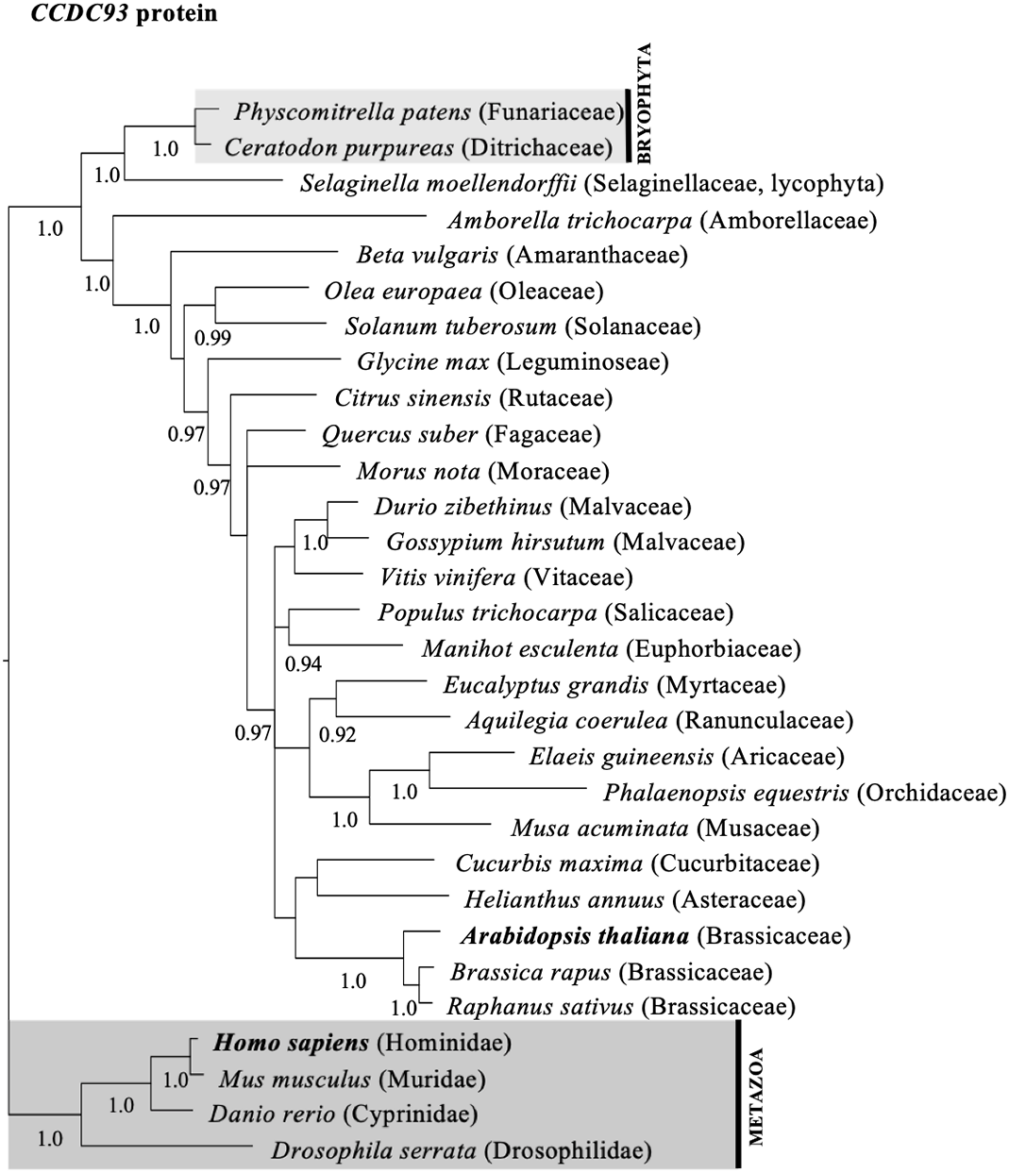
Majority rule consensus tree based on Bayesian phylogenetic analyses of aligned CCDC93 protein sequences. Numbers below branches denote Bayesian posterior probabilities above 0.89. CCDC proteins/genes from Arabidopsis and humans are in bold, and shaded boxes highlight animals (metazoans; dark grey box) and bryophytes (bryophyta; light grey box).

To investigate whether the diversity of CCDC22 and CCDC93 genes in plants shared a similar evolutionary history to VPS26C, we queried genomic and transcriptome databases and generated phylogenetic trees based on either DNA sequences (**Figures S4**, **S5**) or predicted amino acid (**Figures 3**, **4**) sequences. This analysis identified a single ortholog for both CCDC22 and CCDC93 in many eudicots. Phylogenetic analysis also identified an ortholog of CCDC22 and CCDC93 in the monocots *Asparagus officinalis* (Asparagaceae) and/or *Elaeis guineensis* (Aricaceae), *Phalaenopsis equestris* (Orchidaceae), and *Musa acuminata* (Musaceae) (**Figures 3**, **4**). However, CCDC22 and CCDC93 sequences were absent from grass (Poaceae) genomes. These analyses imply that, like *VPS26C*, *CCDC22* and *CCDC93* were lost after the divergence of Poales from Commelinales-Zingiberales, but before the origin of grasses.

### 3.3 CCDC22 and CCDC93 are required for root and root hair growth in Arabidopsis

**Figure 5:**
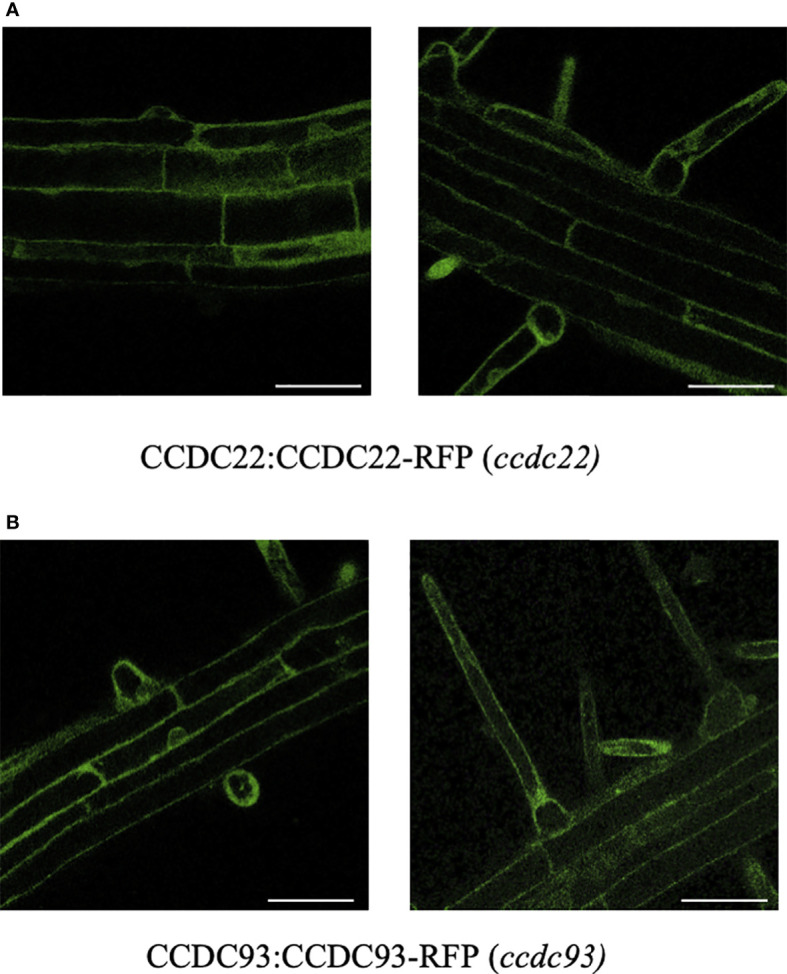
CCDC22-RFP and CCDC93-RFP localize to the cytoplasm of root hairs and root epidermal cells. Confocal fluorescence microscopy was used to visualize **(A)** CCDC22:CCDC22-RFP (ccdc22-1) and **(B)** CCDC93:CCDC93-RFP (ccdc93-1) expression in seedling roots. Scalebars are equal to 50μm.

Arabidopsis seedlings expressing CCDC22:CCDC22-RFP and CCDC93:CCDC93-RFP were generated by first identifying homozygous *ccdc22-1* and *ccdc93-1* mutants and then transforming them with the appropriate CCDC-RFP fusion. Transgenic seedlings homozygous for these RFP fusions were then identified using progeny selection (see methods). The expression of CCDC22-RFP and CCDC93-RFP were analyzed using confocal microscopy to determine the intracellular pattern of accumulation of these proteins in Arabidopsis root hair and epidermal cells. Both CCDC22:CCDC22-RFP and CCDC93:CCDC93-RFP were expressed in seedling roots and were primarily localized to the cytoplasm of root epidermal cells (**Figure 5**).

**Figure 6:**
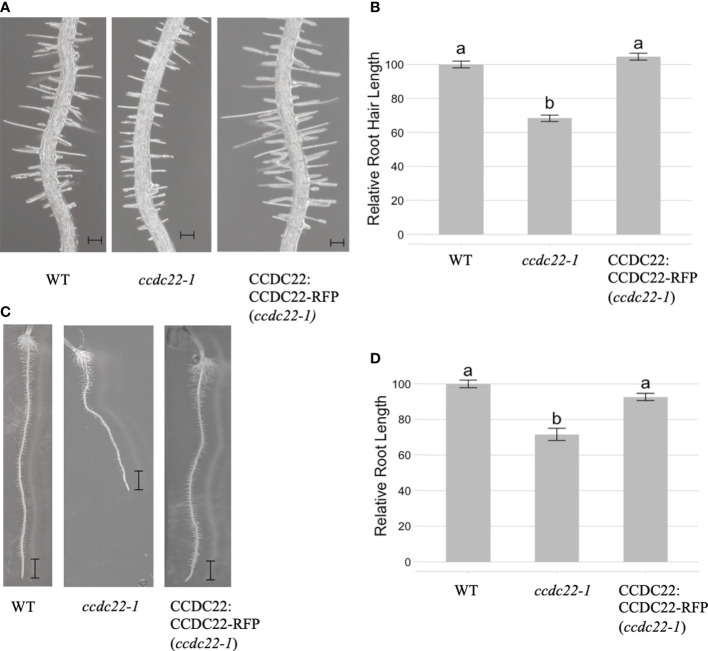
*CCDC22* is required for root and root hair growth in Arabidopsis seedlings. **(A)** Images of the root hairs of five-day old wild type, *ccdc22-1* and *CCDC22:CCDC22-RFP (ccdc22-1)* seedlings grown on 1X MS media, Scale bars are equal to 100 mm. **(B)** Relative root hair lengths of the indicated genotypes as compared to wild type (WT). Represented is the combined data from three independent experiments in which 15 root hairs per seedling and 10 seedlings per genotype were measured. **(C)** Expression of a C-terminal RFP fusion to *CCDC22* in the *ccdc22-1* background resulted in complementation of the *ccdc22-1* short root phenotype. Scalebars are equal to 1mm. **(D)** Relative root growth (%) of the indicated genotypes. Letters above the bars represent statistically significant groupings at the level of *p <*0.001 as determined by analysis of variance (ANOVA) and Tukey’s honestly significance difference tests.

**Figure 7:**
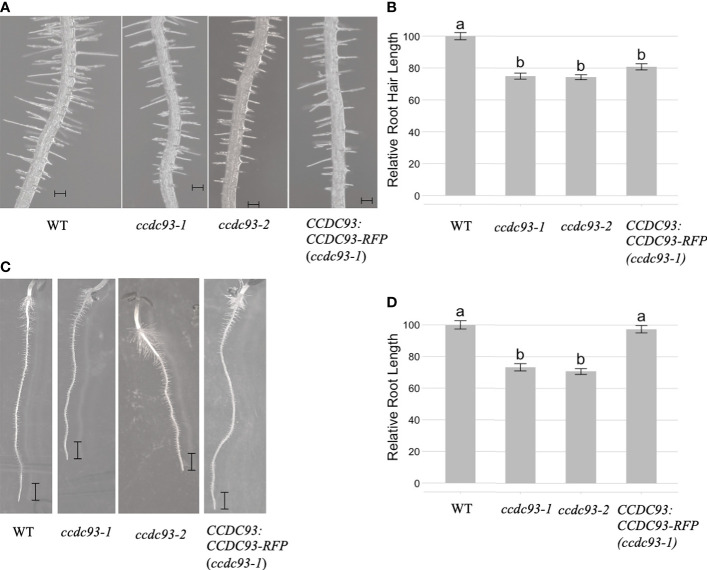
*CCDC93* is required for root and root hair growth in Arabidopsis seedlings. **(A)** Root hairs of five-day-old wild type, *ccdc93-1* and *ccdc93-2* seedlings grown on 1X MS media are shown, Scalebars are equal to 100 μm. **(B)** Quantification of five-day old root hair length of seedlings grown on 1X MS media. Data presented are the pooled results of three independent experiments in which 15 root hairs per seedling and 10 seedlings per genotype were measured. **(C)** Expression of a C-terminal RFP fusion to *CCDC93* in the *ccdc93-1* background resulted in complementation of the *ccdc93-1* short root phenotype. Scalebars are equal to 1mm. **(D)** Relative root growth (%) of the indicated genotypes. Letters above the bars represent statistically significant groupings at the level of *p <*0.001 as determined by analysis of variance (ANOVA) and Tukey’s honestly significance difference tests.

We then investigated whether CCDC22 and/or CCDC93 function in the same developmental pathway as VPS26C by analyzing root hair growth in *ccdc22* and *ccdc93* T-DNA insertion mutants. qRT/PCR of *ccdc22-1*, *ccdc93-1*, and *ccdc93-2* demonstrated that these were null alleles (**Figure S6**). When *ccdc22-1* and wild type seedlings were grown for five days on 1X MS media and analyzed, *ccdc22-1* seedlings were found to have shorter root hairs than wild type seedlings (**Figures 6A, B**). A similar result was observed for five-day-old *ccdc93-1* and *ccdc93-2* seedlings when compared with wild type (**Figures 7A, B**). These data demonstrate that CCDC22 and CCDC93 contribute to pathways necessary for root hair growth in Arabidopsis seedlings.

Analysis of five-day-old *ccdc22* and *ccdc93* seedlings also showed that these proteins are required for root growth in Arabidopsis (**Figures 6C, D** and **Figures 7C, D**). Expression of CCDC22:CCDC22-RFP in the *ccdc22* background complemented both the root hair and root growth defect observed in *ccdc22*, confirming that loss of CCDC22 results in a defect in growth of roots and root hairs (**Figure 6**). Expression of CCDC93:CCDC93-RFP in the *ccdc93* background complemented the root growth phenotype but not the root hair growth phenotype (**Figure 7**). Detection of a root hair growth defect in two independent *ccdc93* alleles (**Figure 7**) supports a role for CCDC93 in pathways required for root hair growth. The lack of complementation of the root hair phenotype in *ccdc93-1* mutants expressing a CCDC93:CCDC93-RFP fusion suggests that the CCDC93-RFP fusion protein may not be functional in this complementation assay due to steric hindrance that may prevent CCDC93 from interacting with other proteins needed to promote root hair growth,.

### 3.4 *ccdc93* is a genetic suppressor of the *vti13* root hair growth phenotype

VTI13, a SNARE required for root hair growth and cell wall organization of root epidermal cells, is localized to early endosomes and the vacuole membrane (Larson et al., 2014). We have previously shown a genetic interaction between the VTI13 trafficking pathway and a cellular pathway involving *VPS26C* as *vps26c* is a genetic suppressor of both the root hair growth and cell wall organization phenotypes of *vti13* (Jha et al., 2018).

**Figure 8:**
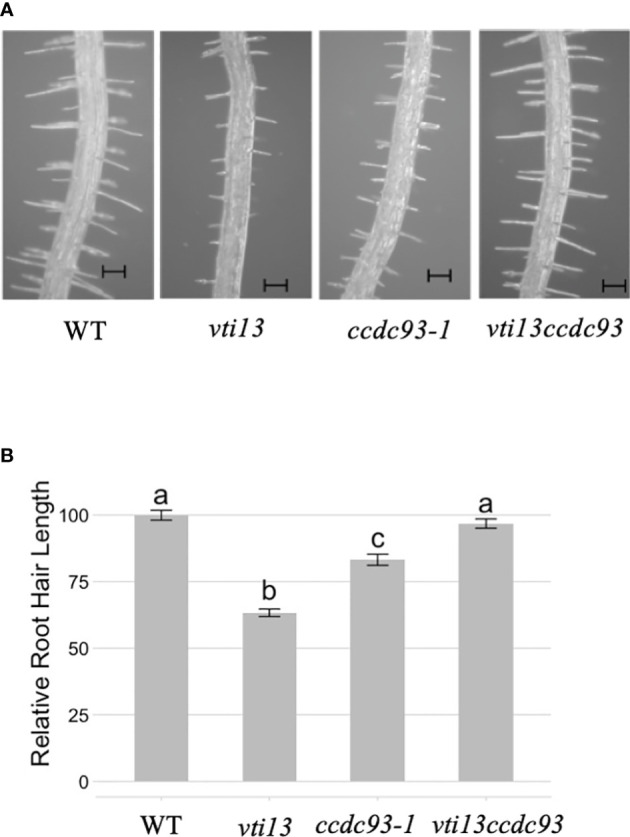
ccdc93-1 suppresses the vti13 short root hair phenotype. **(A)** Representative root hair images of wild type, vti13, ccdc93 and vti13 ccdc93 seedlings after 5 days of growth on 1X MS media. the bars are equal to 100 mm. **(B)** Quantification of five-day-old seedling root hair phenotypes grown on 1X MS media. Data presented are the pooled results of three independent experiments in which 15 root hairs per seedling were measured and 10 total seedlings/genotype were assayed. Letters above the bar represent statistically significant groupings at the p<0.001 level are based upon the results of analysis of variance (ANOVA) and Tukey’s honestly significant difference tests. Scale bars are equal to 100 mm.

To further define the role of proteins that may function with the VPS26C retriever complex, we asked whether *ccdc93* could also act as a genetic suppressor of the *vti13* root hair phenotype. To test this, we compared root hair lengths of *ccdc93* and *vti13 ccdc93* seedlings with wild type and *vti13* seedlings. Analysis of *vti13 ccdc93* root hair length demonstrated that it was similar to that of wild type seedlings (**Figure 8**). These results support a role for CCDC93 in a pathway that genetically interacts with the VTI13 trafficking pathway to the lytic vacuole and provide evidence that CCDC93 may function with the VPS26C retriever-like complex in regulating the growth of root hairs.

## 4 Discussion

In human cells, CCDC22 and CCDC93 oligomerize with Copper Metabolism MURR1 Domain-containing (COMMD) proteins to form the COMMD/CCDC22/CCDC93 (CCC) complex. This complex interacts with retriever to facilitate the trafficking of roughly 120 endocytosed integral plasma membrane proteins from the early endosome back to the plasma membrane (McNally et al., 2017). We have previously demonstrated that a complex homologous to retriever is formed *in planta* and is composed of VPS35A (AT2G17790), VPS29 (AT3G47810), and VPS26C (AT1G48550) (Jha et al., 2018). To date, the only homologues of proteins within the CCC complex that have been identified in flowering plants based on sequence similarity are CCDC22 and CCDC93. The goal of this study was to characterize CCDC22 and CCDC93 in Arabidopsis as a first step in determining whether these proteins function in collaboration with the retriever complex in plants.

### 4.1 CCDC22 and CCDC93 are required for root and root hair growth

We previously demonstrated that mutations in *VPS35A*, *VPS29* and *VPS26C*, genes that encode retriever subunits in Arabidopsis, exhibit short root hair phenotypes implicating retriever in polarized growth. Therefore, when we identified Arabidopsis orthologs of CCDC22 (AT1G55830) and CCDC93 (AT4G32560), we investigated whether their function was also necessary for root hair growth. As predicted, *ccdc22* and *ccdc93* null mutants displayed short root hair phenotypes (**Figures 6**, **7**), with *CCDC22:CCDC22-RFP* complementing the *ccdc22* phenotype. Although the *ccdc93-1* allele was not similarly complemented by expression of an RFP fusion to CCDC93, these data are consistent with a model in which *CCDC22* and *CCDC93* are integrated into a retriever-mediated polarized growth pathway. Failure of complementation in the *ccdc93* background might be explained by steric hindrance of the chimeric protein preventing important protein-protein interactions required for root hair growth as CCDC93:CCDC93-RFP is expressed in root epidermal cells and root hairs (**Figure 5**). Future studies will be needed to test this hypothesis.

In addition to short root hairs, *ccdc22* and *ccdc93* mutants also had roots that are significantly shorter than wild type seedlings after five days of growth on 1X MS media (**Figures 6**, **7**). Expression of *CCDC22:CCDC22-RFP* or *CCDC93:CCDC93-RFP* in their respective mutant backgrounds resulted in the complementation of the short root phenotypes and allowed for the visualization of the intracellular localization and expression pattern of these proteins. As expected from the electronic fluorescent pictographs available through The Bio-Analytic Resource for Plant Biology (bar.utoronto.ca), expression of both CCDC22 and CCDC93 was observed in root hairs, root epidermal cells, and throughout the cells of the root. Both proteins exhibited diffuse, cytosolic localization patterns.

### 4.2 Degeneration of the retriever complex in grasses

Phylogenetic analyses of CCDC22, CCDC93, and VPS26C orthologs in flowering plants indicate that these proteins have similar evolutionary histories regarding gene duplication and loss (**Figures 3**, **4**, **S8**, **S9**). Each gene clearly has an ancient origin as evidenced by its presence across major clades of eukaryotes. However, lineage-specific losses in both animals and plants suggest that these genes are not required in all taxa, and at least in grasses, show a pattern of coordinated loss. Similar examples of continued pathway degeneration following initial gene loss have been found for other traits and species in plants (Preston et al., 2011). Future work will be required to establish how discrete the retriever complex is in plants and to determine whether an alternative complex exists in grasses.

### 4.3 Genetic interactions between pathways involving *VTI11 and VTI13* and those involving the retromer/retriever in plants

VTI11 and VTI13 are SNARE proteins that function in trafficking pathways to the lytic vacuole in Arabidopsis (Sanderfoot et al., 2001; Ebine et al., 2008; Larson et al., 2014). Previous studies have shown that mutations in the retromer subunits *VPS26A* and *VPS35A* are able to suppress the *vti11* shoot agravitropic phenotype (Hashiguchi et al., 2010). Similarly, mutations in the retriever subunit *VPS26C* suppress both the root hair growth and cell wall organization phenotype associated with *vti13* (Jha et al., 2018). We have also shown in this study that a *vti13 ccdc93* double mutant is able suppress the *vti13* root hair phenotype (**Figure 8**). Taken together, these data suggest a genetic interaction between trafficking pathways to the lytic vacuole and endosomal trafficking pathways involving the large retromer/retriever complex in Arabidopsis. While the mechanism associated with the suppression of *vti11* and *vti13* phenotypes has not yet been identified, it is possible that cellular homeostasis requires a balance between the pathways trafficking cargo to the lytic vacuole and the pathways involved in recycling of endosomal proteins to the TGN and/or Golgi. Further work will be required to identify whether a common cellular mechanism is responsible for the genetic suppression of *vti11* and *vti13* phenotypes by mutations in VPS26A, VPS26C, VPS35A and CCDC93 in plants.

## 5 Conclusion

We have identified phenotypes associated with loss of function mutants of CCDC22 and CCDC93, two proteins hypothesized to function in a pathway with the retriever complex in Arabidopsis. In support of this hypothesis, null mutations associated with both proteins have similar phenotypes which are reminiscent of the short root hair phenotypes of retriever mutants. Furthermore, the suppression of the short root hair phenotype associated with VTI13, a SNARE involved in a vacuolar trafficking pathway, by both *vps26c*, a retriever component, and *ccdc93* suggests a functional relationship between CCDC93 and the retriever complex in Arabidopsis. Future experiments will be needed to identify proteins that physically interact with CCDC22 and CCDC93 in Arabidopsis and to establish the role of these proteins in endomembrane trafficking in plants.

## Data availability statement

The original contributions presented in the study are included in the article/**Supplementary Materials**. Further inquiries can be directed to the corresponding author.

## Author contributions

CL performed all experiments except for the phylogenetic analysis. JP performed the phylogenetic analysis and wrote the methods for this as well as constructed the phylogenies. MT wrote the introduction and the results section. CL wrote the methods and Discussion sections. JP wrote a portion of both the results and discussion pertaining to the phylogenetic relationships discussed in paper. All authors contributed to the article and approved the submitted version.

## Supplementary material

Supplementary Figure 1 Relative expression of *CCDC22* and *CCDC93* in wild type (WT) and the *vps26c-1* mutant background. RNA was extracted from 5-day old seedlings grown on 1X MS media. First strand cDNA was generated and used as template for qRT/PCR. Expression levels were normalized to WT levels of *ACT2* expression and compared using a student’s t-test. *p value < 0.05; *** p value <0.001.

Supplementary Figure 2 Predicted Amino Acid Sequence of CCDC22 derived from the cDNA sequence of CCDC22. The full-length coding sequence of CCDC22 was determined by sequencing PCR products amplified from first strand cDNA using the primers ccdc22_pENTR_F and ccdc22_R_Stop.

Supplementary Figure 3 Predicted Amino Acid sequence of CCDC93 derived from the cDNA sequence of CCDC93. The full-length coding sequence of CCDC93 was determined by sequencing PCR products amplified from first strand cDNA using the primers ccdc93_pENTR_F and ccdc93_R_Stop.

Supplementary Figure 4 Majority rule consensus tree based on Bayesian phylogenetic analyses of aligned *CCDC22* coding region nucleotide sequences. Numbers below branches denote Bayesian posterior probabilities above 0.89. Arabidopsis *CCDC22* is indicated in bold.

Supplementary Figure 5 Majority rule consensus tree based on Bayesian phylogenetic analyses of aligned *CCDC93* coding region nucleotide sequences. Numbers below branches denote Bayesian posterior probabilities above 0.89. Arabidopsis *CCDC93* is indicated in bold.

Supplementary Figure 6 CCDC22 and CCDC93 expression levels in WT and their respective mutant backgrounds. RNA was extracted from 7-day old seedlings grown on 1X MS media. First strand cDNA was generated and used as template for qRT/PCR. Primers ccdc22_qRT1_F and ccdc22qRT1_R were used to quantitate level of CCDC22 transcript and primers ccdc93_qRT1_F and ccdc93qRT1_R were used to quantitate the level of CCDC93 transcript in wild type and mutant backgrounds. Expression levels were normalized to WT levels of ACT2 expression and compared using a student’s t-test. *p value < 0.05; *** p value <0.001.

Supplementary Figure 7 Genotyping of *ccdc22-1* and the CCDC22:CCDC22-RFP (ccdc22-1) transgenic line. **(A)** Visualization of the amplification of PCR products from isolated genomic DNA in which the following primer combinations were used: Primer 1) ccdc22_pENTR_F/ccdc22_R_STOP 2) LB1.3/ccdc22_pENTR_F 3) 22proSacIpENTR_F/RFP_R. See **Table S1** for primer sequences. **(B)** Gene model for the transgenic RFP fusion construct with the locations of the indicated primer pairs. Image is drawn to scale.

Supplementary Figure 8 Genotyping of *ccdc93-1* and the CCDC93:CCDC93-RFP (ccdc93-1) transgenic line. **(A)** Visualization of the amplification of PCR products from isolated genomic DNA in which the following primer combinations were used: 1) ccdc93_pENTR_F/ccdc93_R_STOP; 2) ccdc93_pENTR_F/LB1.3; 3) 93proSacIpENTR_F/RFP_R. See **Table S1** for the primer sequences. **(B)** Gene model for the transgenic RFP fusion construct with the locations of the indicated primer pairs. Image is drawn to scale.

Supplementary Figure 9 Genotyping *ccdc93-2* line. Primer combinations are: 1) ccdc93_pENTR_F/ccdc93_R_STOP and 2) ccdc93_pENTR_F/LB1.3. (7) 5-day old seedlings were used per *ccdc93-2* genomic DNA preparation.

Supplementary Figure 10 Genotyping of *vti13*, *ccdc93-1*, and *vti13ccdc93-1*. The primer combinations are: 1) vti13_dtopo_F/vti13_dtopo_R; 2) vti13_dtopo_F/LB1.3; 3) ccdc93_pENTR_F/ccdc93_R_STOP; 4) ccdc93_pENTR_F/LB1.3. See **Table S1** for primer sequences.
