# Supplementary figures and images for: CCDC22 and CCDC93, two potential retriever-interacting proteins, are required for root and root hair growth in Arabidopsis
Source: Front Plant Sci. 2022 Dec 22;13:1051503. doi: 10.3389/fpls.2022.1051503 (PMC9815543; doi:10.3389/fpls.2022.1051503)

## Slide 1
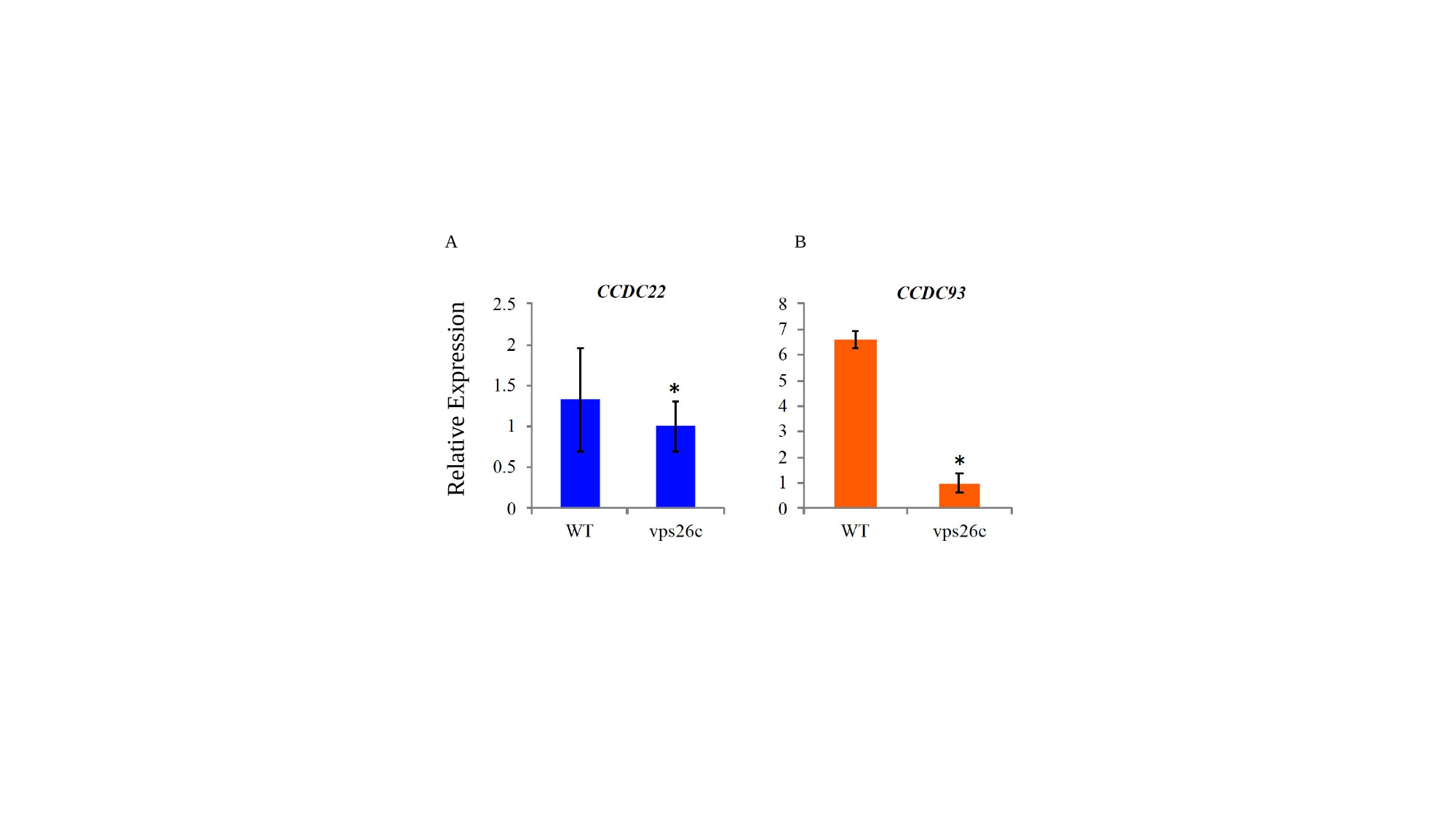

A
B
Relative Expression

Supplement: Supplementary Figure 1 — Relative expression of CCDC22 and CCDC93 in wild type (WT) and the vps26c-1 mutant background. RNA was extracted from 5-day old seedlings grown on 1X MS media. First strand cDNA was generated and used as template for qRT/PCR. Expression levels were normalized to WT levels of ACT2 expression and compared using a student’s t-test. *p value < 0.05; *** p value <0.001. [file Presentation_1.pptx]

## Slide 1
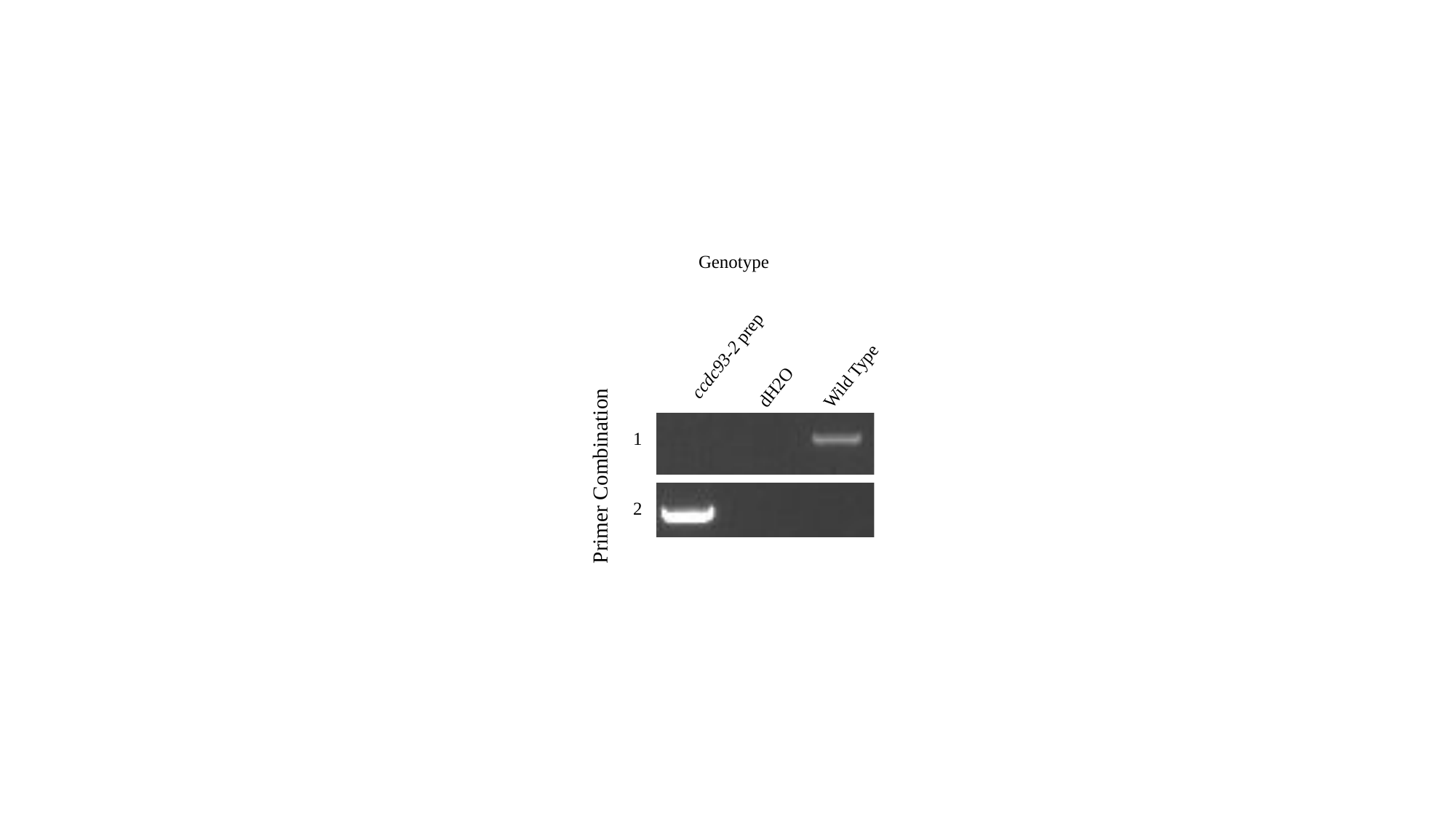

Genotype
ccdc93-2 prep
Wild Type
dH2O
1
Primer Combination
2

Supplement: Supplementary Figure 9 — Genotyping ccdc93-2 line. Primer combinations are: 1) ccdc93_pENTR_F/ccdc93_R_STOP and 2) ccdc93_pENTR_F/LB1.3. (7) 5-day old seedlings were used per ccdc93-2 genomic DNA preparation. [file Presentation_9.pptx]

## Slide 1
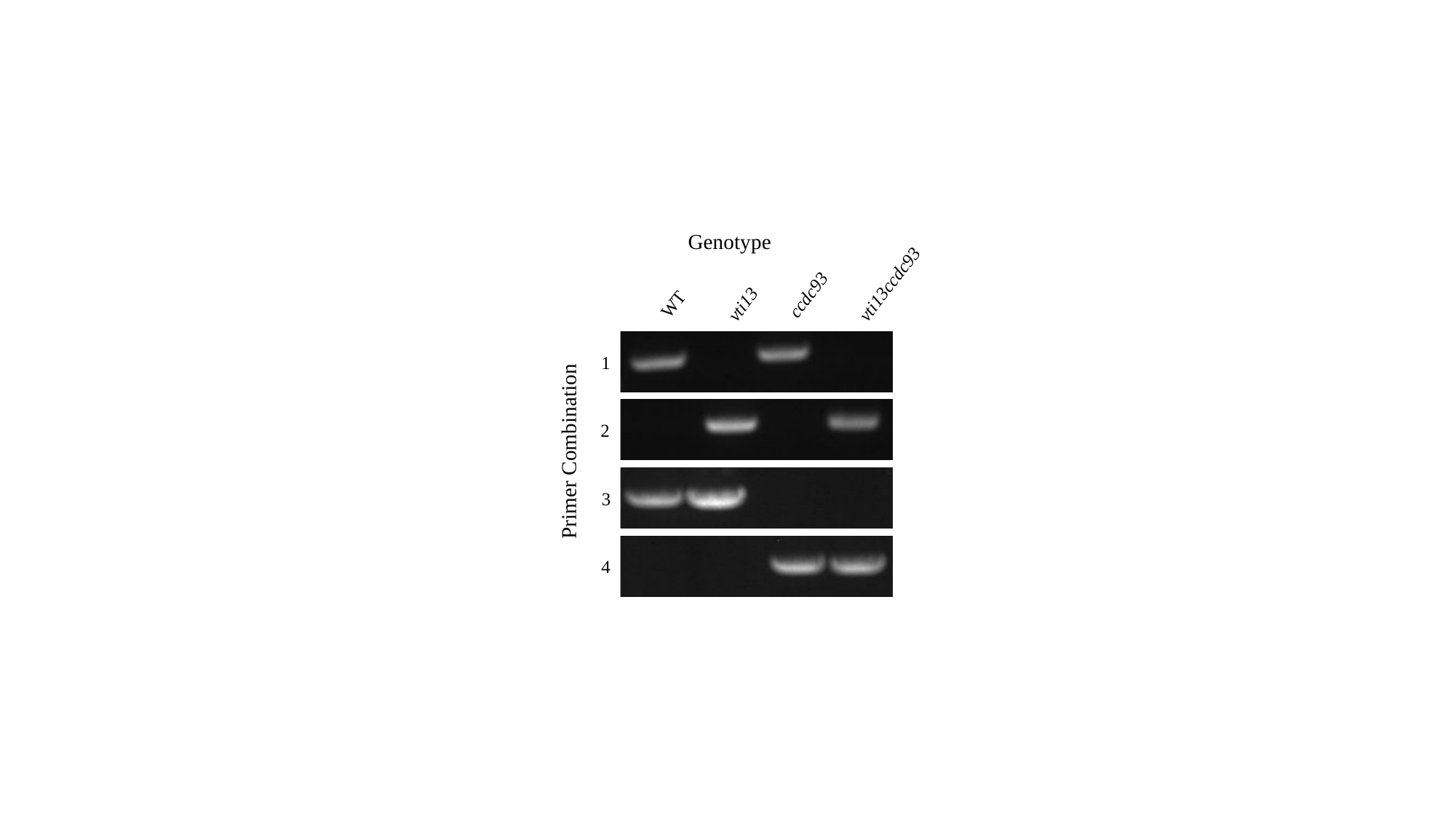

Genotype
vti13ccdc93
ccdc93
WT
vti13
1
2
Primer Combination
3
4

Supplement: Supplementary Figure 10 — Genotyping of vti13, ccdc93-1, and vti13ccdc93-1. The primer combinations are: 1) vti13_dtopo_F/vti13_dtopo_R; 2) vti13_dtopo_F/LB1.3; 3) ccdc93_pENTR_F/ccdc93_R_STOP; 4) ccdc93_pENTR_F/LB1.3. See Table S1 for primer sequences. [file Presentation_10.pptx]
