# Supplementary material for: CCDC22 and CCDC93, two potential retriever-interacting proteins, are required for root and root hair growth in Arabidopsis
Source: Front Plant Sci. 2022 Dec 22;13:1051503. doi: 10.3389/fpls.2022.1051503 (PMC9815543; doi:10.3389/fpls.2022.1051503)
Supplement: Supplementary Figure 2 — Predicted Amino Acid Sequence of CCDC22 derived from the cDNA sequence of CCDC22. The full-length coding sequence of CCDC22 was determined by sequencing PCR products amplified from first strand cDNA using the primers ccdc22_pENTR_F and ccdc22_R_Stop. [file Presentation_2.pptx]

## Slide 1
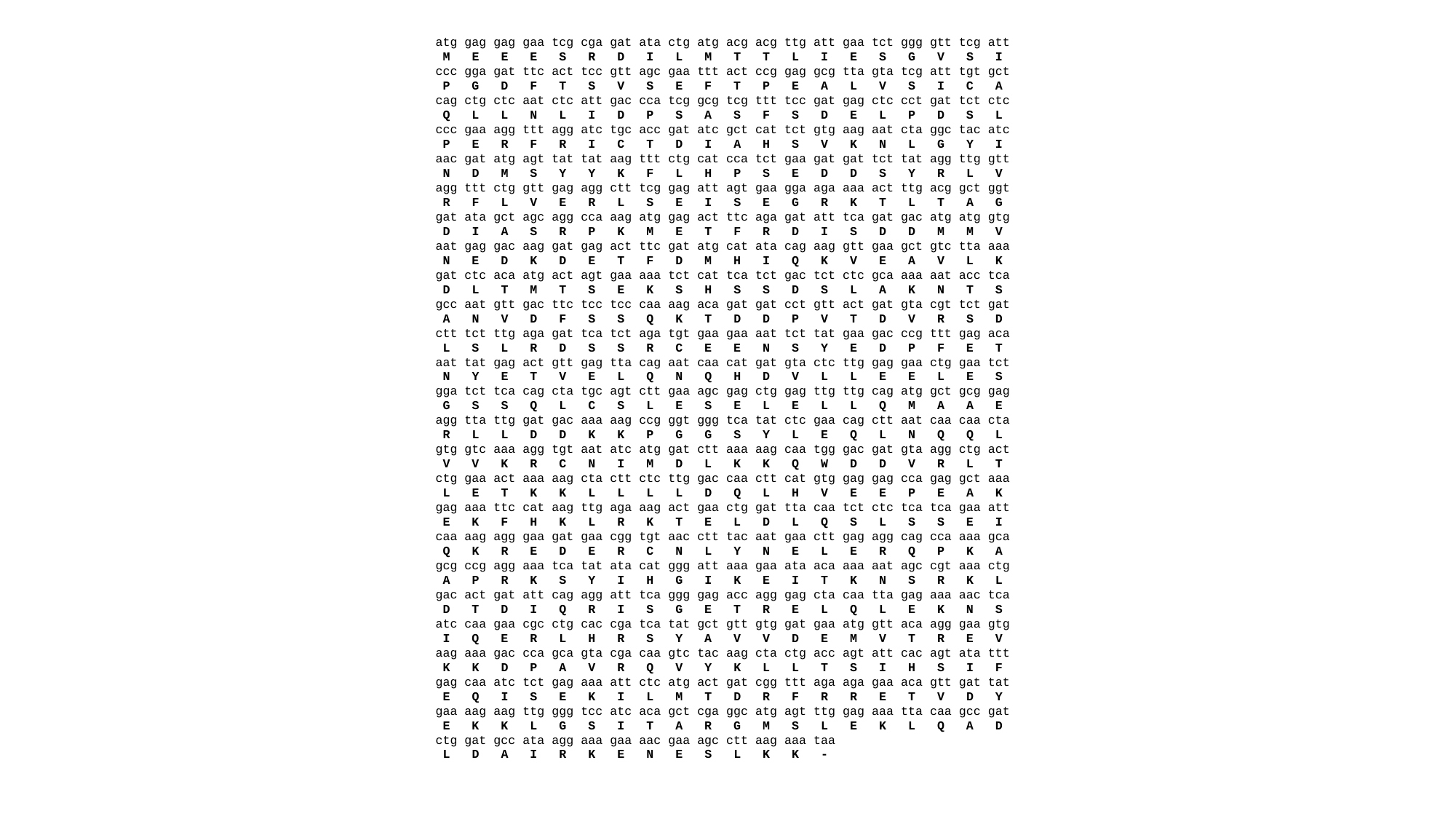

atg gag gag gaa tcg cga gat ata ctg atg acg acg ttg att gaa tct ggg gtt tcg att 
 M   E   E   E   S   R   D   I   L   M   T   T   L   I   E   S   G   V   S   I  
ccc gga gat ttc act tcc gtt agc gaa ttt act ccg gag gcg tta gta tcg att tgt gct 
 P   G   D   F   T   S   V   S   E   F   T   P   E   A   L   V   S   I   C   A  
cag ctg ctc aat ctc att gac cca tcg gcg tcg ttt tcc gat gag ctc cct gat tct ctc 
 Q   L   L   N   L   I   D   P   S   A   S   F   S   D   E   L   P   D   S   L  
ccc gaa agg ttt agg atc tgc acc gat atc gct cat tct gtg aag aat cta ggc tac atc 
 P   E   R   F   R   I   C   T   D   I   A   H   S   V   K   N   L   G   Y   I  
aac gat atg agt tat tat aag ttt ctg cat cca tct gaa gat gat tct tat agg ttg gtt 
 N   D   M   S   Y   Y   K   F   L   H   P   S   E   D   D   S   Y   R   L   V  
agg ttt ctg gtt gag agg ctt tcg gag att agt gaa gga aga aaa act ttg acg gct ggt 
 R   F   L   V   E   R   L   S   E   I   S   E   G   R   K   T   L   T   A   G  
gat ata gct agc agg cca aag atg gag act ttc aga gat att tca gat gac atg atg gtg 
 D   I   A   S   R   P   K   M   E   T   F   R   D   I   S   D   D   M   M   V  
aat gag gac aag gat gag act ttc gat atg cat ata cag aag gtt gaa gct gtc tta aaa 
 N   E   D   K   D   E   T   F   D   M   H   I   Q   K   V   E   A   V   L   K  
gat ctc aca atg act agt gaa aaa tct cat tca tct gac tct ctc gca aaa aat acc tca 
 D   L   T   M   T   S   E   K   S   H   S   S   D   S   L   A   K   N   T   S  
gcc aat gtt gac ttc tcc tcc caa aag aca gat gat cct gtt act gat gta cgt tct gat 
 A   N   V   D   F   S   S   Q   K   T   D   D   P   V   T   D   V   R   S   D  
ctt tct ttg aga gat tca tct aga tgt gaa gaa aat tct tat gaa gac ccg ttt gag aca 
 L   S   L   R   D   S   S   R   C   E   E   N   S   Y   E   D   P   F   E   T  
aat tat gag act gtt gag tta cag aat caa cat gat gta ctc ttg gag gaa ctg gaa tct 
 N   Y   E   T   V   E   L   Q   N   Q   H   D   V   L   L   E   E   L   E   S  
gga tct tca cag cta tgc agt ctt gaa agc gag ctg gag ttg ttg cag atg gct gcg gag 
 G   S   S   Q   L   C   S   L   E   S   E   L   E   L   L   Q   M   A   A   E  
agg tta ttg gat gac aaa aag ccg ggt ggg tca tat ctc gaa cag ctt aat caa caa cta 
 R   L   L   D   D   K   K   P   G   G   S   Y   L   E   Q   L   N   Q   Q   L  
gtg gtc aaa agg tgt aat atc atg gat ctt aaa aag caa tgg gac gat gta agg ctg act 
 V   V   K   R   C   N   I   M   D   L   K   K   Q   W   D   D   V   R   L   T  
ctg gaa act aaa aag cta ctt ctc ttg gac caa ctt cat gtg gag gag cca gag gct aaa 
 L   E   T   K   K   L   L   L   L   D   Q   L   H   V   E   E   P   E   A   K  
gag aaa ttc cat aag ttg aga aag act gaa ctg gat tta caa tct ctc tca tca gaa att 
 E   K   F   H   K   L   R   K   T   E   L   D   L   Q   S   L   S   S   E   I  
caa aag agg gaa gat gaa cgg tgt aac ctt tac aat gaa ctt gag agg cag cca aaa gca 
 Q   K   R   E   D   E   R   C   N   L   Y   N   E   L   E   R   Q   P   K   A  
gcg ccg agg aaa tca tat ata cat ggg att aaa gaa ata aca aaa aat agc cgt aaa ctg 
 A   P   R   K   S   Y   I   H   G   I   K   E   I   T   K   N   S   R   K   L  
gac act gat att cag agg att tca ggg gag acc agg gag cta caa tta gag aaa aac tca 
 D   T   D   I   Q   R   I   S   G   E   T   R   E   L   Q   L   E   K   N   S  
atc caa gaa cgc ctg cac cga tca tat gct gtt gtg gat gaa atg gtt aca agg gaa gtg 
 I   Q   E   R   L   H   R   S   Y   A   V   V   D   E   M   V   T   R   E   V  
aag aaa gac cca gca gta cga caa gtc tac aag cta ctg acc agt att cac agt ata ttt 
 K   K   D   P   A   V   R   Q   V   Y   K   L   L   T   S   I   H   S   I   F  
gag caa atc tct gag aaa att ctc atg act gat cgg ttt aga aga gaa aca gtt gat tat 
 E   Q   I   S   E   K   I   L   M   T   D   R   F   R   R   E   T   V   D   Y  
gaa aag aag ttg ggg tcc atc aca gct cga ggc atg agt ttg gag aaa tta caa gcc gat 
 E   K   K   L   G   S   I   T   A   R   G   M   S   L   E   K   L   Q   A   D  
ctg gat gcc ata agg aaa gaa aac gaa agc ctt aag aaa taa 
 L   D   A   I   R   K   E   N   E   S   L   K   K   -
