# Supplementary material for: CCDC22 and CCDC93, two potential retriever-interacting proteins, are required for root and root hair growth in Arabidopsis
Source: Front Plant Sci. 2022 Dec 22;13:1051503. doi: 10.3389/fpls.2022.1051503 (PMC9815543; doi:10.3389/fpls.2022.1051503)
Supplement: Supplementary Figure 3 — Predicted Amino Acid sequence of CCDC93 derived from the cDNA sequence of CCDC93. The full-length coding sequence of CCDC93 was determined by sequencing PCR products amplified from first strand cDNA using the primers ccdc93_pENTR_F and ccdc93_R_Stop. [file Presentation_3.pptx]

## Slide 1
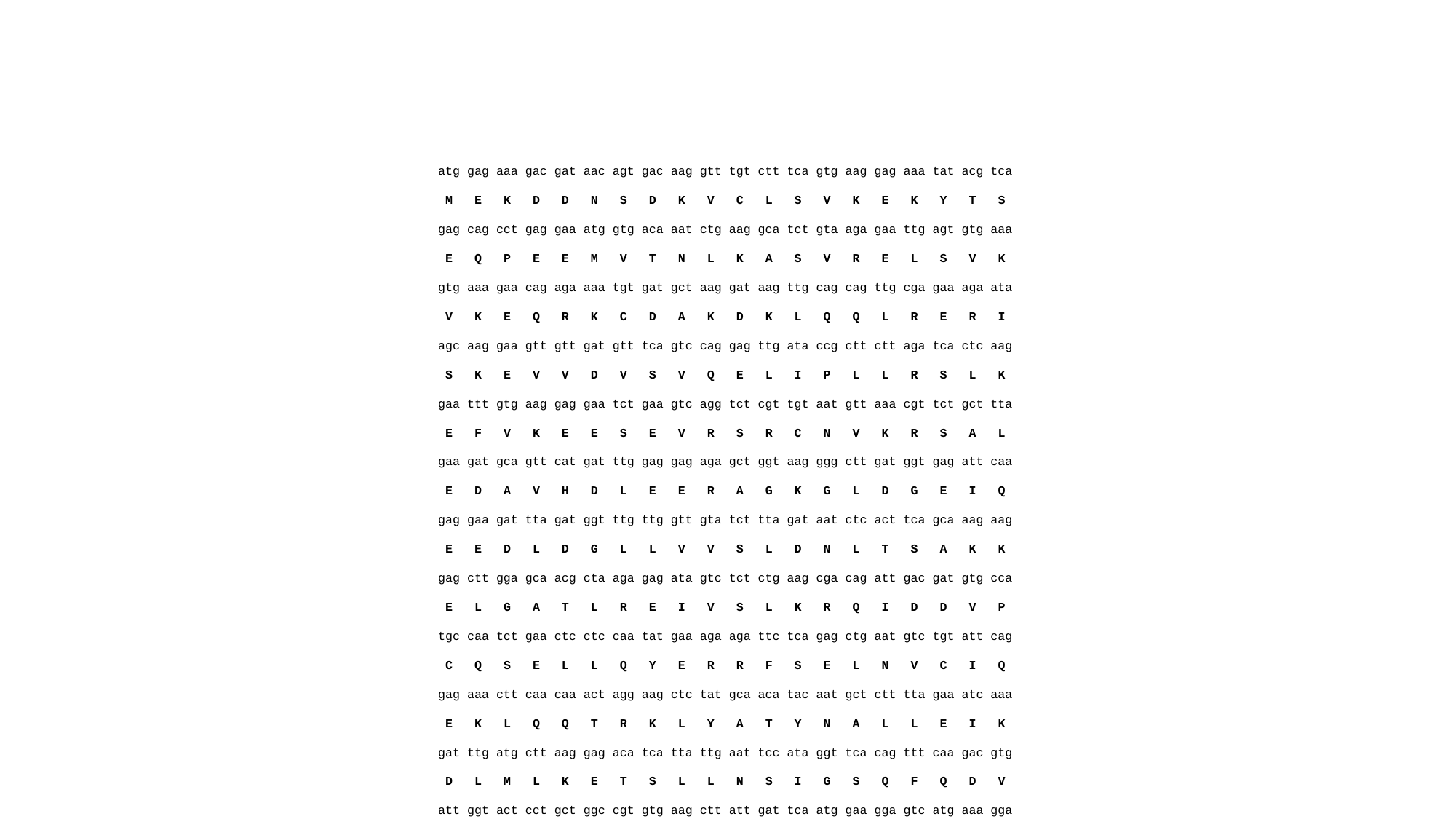

atg gag aaa gac gat aac agt gac aag gtt tgt ctt tca gtg aag gag aaa tat acg tca 
 M   E   K   D   D   N   S   D   K   V   C   L   S   V   K   E   K   Y   T   S  
gag cag cct gag gaa atg gtg aca aat ctg aag gca tct gta aga gaa ttg agt gtg aaa 
 E   Q   P   E   E   M   V   T   N   L   K   A   S   V   R   E   L   S   V   K  
gtg aaa gaa cag aga aaa tgt gat gct aag gat aag ttg cag cag ttg cga gaa aga ata 
 V   K   E   Q   R   K   C   D   A   K   D   K   L   Q   Q   L   R   E   R   I  
agc aag gaa gtt gtt gat gtt tca gtc cag gag ttg ata ccg ctt ctt aga tca ctc aag 
 S   K   E   V   V   D   V   S   V   Q   E   L   I   P   L   L   R   S   L   K  
gaa ttt gtg aag gag gaa tct gaa gtc agg tct cgt tgt aat gtt aaa cgt tct gct tta 
 E   F   V   K   E   E   S   E   V   R   S   R   C   N   V   K   R   S   A   L  
gaa gat gca gtt cat gat ttg gag gag aga gct ggt aag ggg ctt gat ggt gag att caa 
 E   D   A   V   H   D   L   E   E   R   A   G   K   G   L   D   G   E   I   Q  
gag gaa gat tta gat ggt ttg ttg gtt gta tct tta gat aat ctc act tca gca aag aag 
 E   E   D   L   D   G   L   L   V   V   S   L   D   N   L   T   S   A   K   K  
gag ctt gga gca acg cta aga gag ata gtc tct ctg aag cga cag att gac gat gtg cca 
 E   L   G   A   T   L   R   E   I   V   S   L   K   R   Q   I   D   D   V   P  
tgc caa tct gaa ctc ctc caa tat gaa aga aga ttc tca gag ctg aat gtc tgt att cag 
 C   Q   S   E   L   L   Q   Y   E   R   R   F   S   E   L   N   V   C   I   Q  
gag aaa ctt caa caa act agg aag ctc tat gca aca tac aat gct ctt tta gaa atc aaa 
 E   K   L   Q   Q   T   R   K   L   Y   A   T   Y   N   A   L   L   E   I   K  
gat ttg atg ctt aag gag aca tca tta ttg aat tcc ata ggt tca cag ttt caa gac gtg 
 D   L   M   L   K   E   T   S   L   L   N   S   I   G   S   Q   F   Q   D   V  
att ggt act cct gct ggc cgt gtg aag ctt att gat tca atg gaa gga gtc atg aaa gga 
 I   G   T   P   A   G   R   V   K   L   I   D   S   M   E   G   V   M   K   G  
atc caa cag aag att gga aaa ata caa ctt ggg ctt caa gaa gag cag agg ctc cgt gat 
 I   Q   Q   K   I   G   K   I   Q   L   G   L   Q   E   E   Q   R   L   R   D  
gct tca aaa gaa aag tat ata gct gca gct gca gag caa aga aaa tgc tac act gta cta 
 A   S   K   E   K   Y   I   A   A   A   A   E   Q   R   K   C   Y   T   V   L  
aga gca tac cag gag gaa tgt act aag aat gag agg ctg aga agt cat ata tct gcc atg 
 R   A   Y   Q   E   E   C   T   K   N   E   R   L   R   S   H   I   S   A   M  
aac gaa cac ctc tga 
 N   E   H   L   -
