# Supplementary material for: CCDC22 and CCDC93, two potential retriever-interacting proteins, are required for root and root hair growth in Arabidopsis
Source: Front Plant Sci. 2022 Dec 22;13:1051503. doi: 10.3389/fpls.2022.1051503 (PMC9815543; doi:10.3389/fpls.2022.1051503)
Supplement: Supplementary Figure 4 — Majority rule consensus tree based on Bayesian phylogenetic analyses of aligned CCDC22 coding region nucleotide sequences. Numbers below branches denote Bayesian posterior probabilities above 0.89. Arabidopsis CCDC22 is indicated in bold. [file Presentation_4.pptx]

## Slide 1
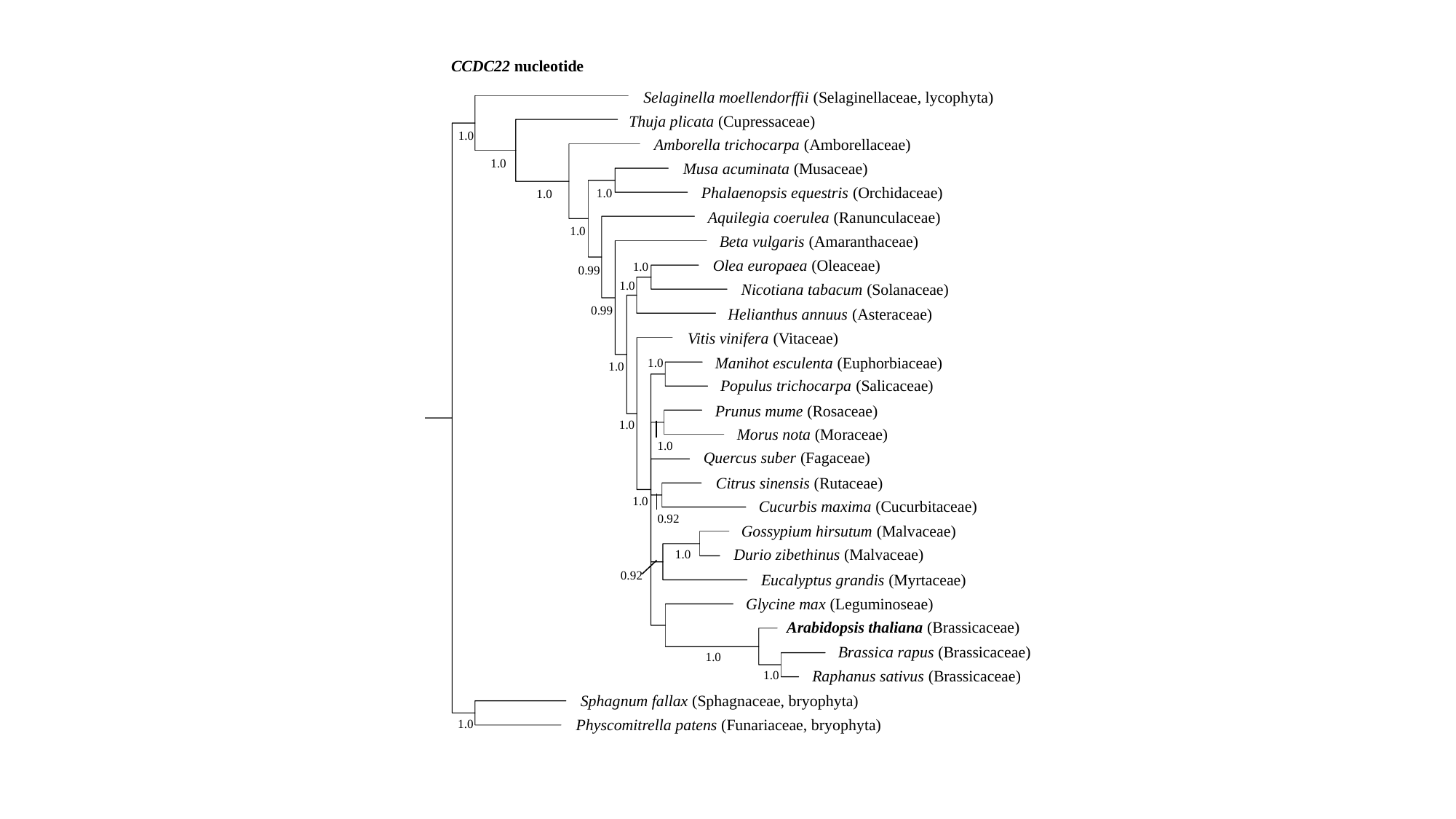

CCDC22 nucleotide
Selaginella moellendorffii (Selaginellaceae, lycophyta)
Thuja plicata (Cupressaceae)
1.0
Amborella trichocarpa (Amborellaceae)
1.0
Musa acuminata (Musaceae)
Phalaenopsis equestris (Orchidaceae)
1.0
1.0
Aquilegia coerulea (Ranunculaceae)
1.0
Beta vulgaris (Amaranthaceae)
Olea europaea (Oleaceae)
1.0
0.99
1.0
Nicotiana tabacum (Solanaceae)
0.99
Helianthus annuus (Asteraceae)
Vitis vinifera (Vitaceae)
Manihot esculenta (Euphorbiaceae)
1.0
1.0
Populus trichocarpa (Salicaceae)
Prunus mume (Rosaceae)
1.0
Morus nota (Moraceae)
1.0
Quercus suber (Fagaceae)
Citrus sinensis (Rutaceae)
1.0
Cucurbis maxima (Cucurbitaceae)
0.92
Gossypium hirsutum (Malvaceae)
Durio zibethinus (Malvaceae)
1.0
0.92
Eucalyptus grandis (Myrtaceae)
Glycine max (Leguminoseae)
Arabidopsis thaliana (Brassicaceae)
Brassica rapus (Brassicaceae)
1.0
Raphanus sativus (Brassicaceae)
1.0
Sphagnum fallax (Sphagnaceae, bryophyta)
Physcomitrella patens (Funariaceae, bryophyta)
1.0
