# Supplementary material for: CCDC22 and CCDC93, two potential retriever-interacting proteins, are required for root and root hair growth in Arabidopsis
Source: Front Plant Sci. 2022 Dec 22;13:1051503. doi: 10.3389/fpls.2022.1051503 (PMC9815543; doi:10.3389/fpls.2022.1051503)
Supplement: Supplementary Figure 5 — Majority rule consensus tree based on Bayesian phylogenetic analyses of aligned CCDC93 coding region nucleotide sequences. Numbers below branches denote Bayesian posterior probabilities above 0.89. Arabidopsis CCDC93 is indicated in bold. [file Presentation_5.pptx]

## Slide 1
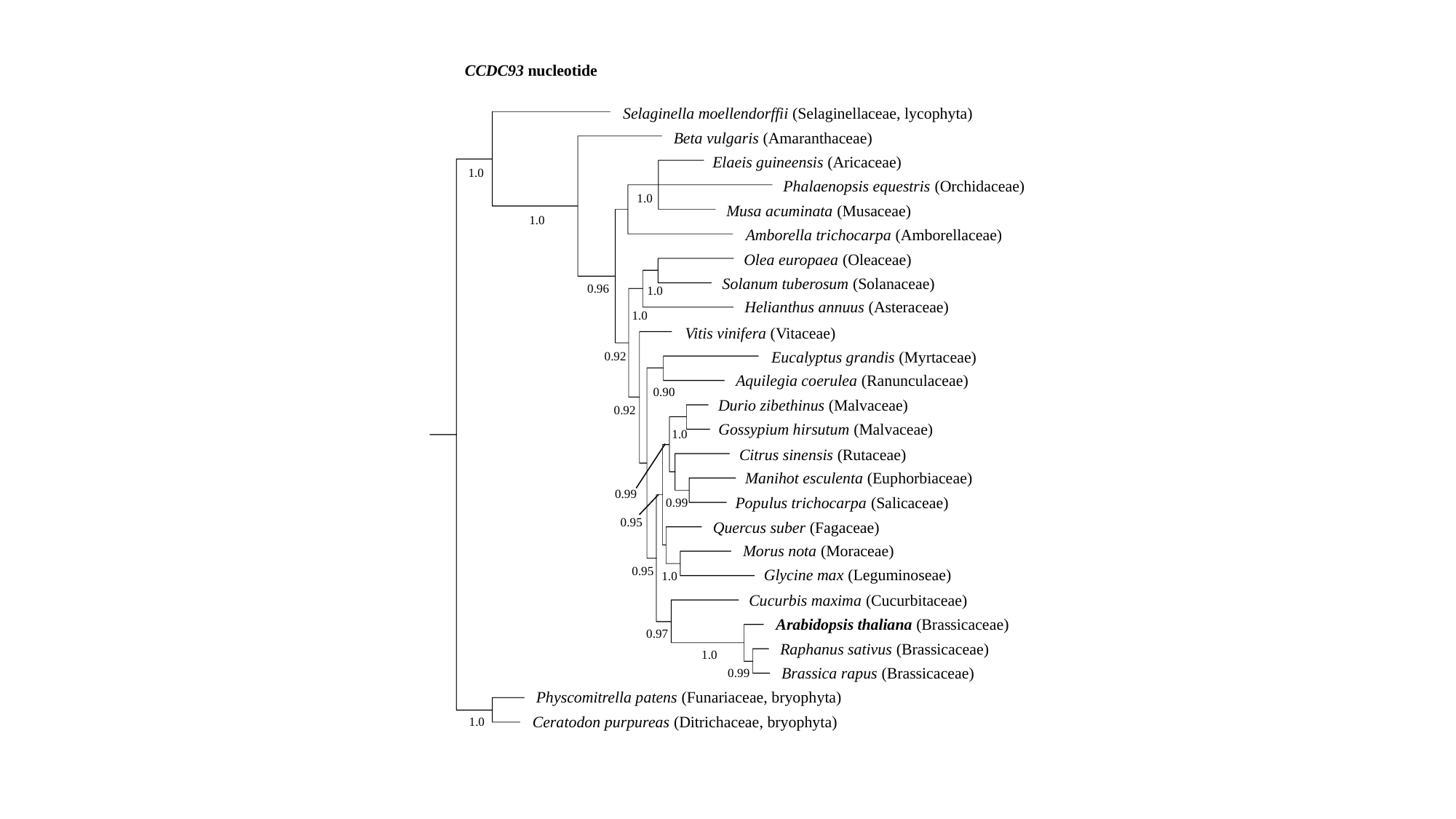

CCDC93 nucleotide
Selaginella moellendorffii (Selaginellaceae, lycophyta)
Beta vulgaris (Amaranthaceae)
Elaeis guineensis (Aricaceae)
1.0
Phalaenopsis equestris (Orchidaceae)
1.0
Musa acuminata (Musaceae)
1.0
Amborella trichocarpa (Amborellaceae)
Olea europaea (Oleaceae)
Solanum tuberosum (Solanaceae)
0.96
1.0
Helianthus annuus (Asteraceae)
1.0
Vitis vinifera (Vitaceae)
Eucalyptus grandis (Myrtaceae)
0.92
Aquilegia coerulea (Ranunculaceae)
0.90
Durio zibethinus (Malvaceae)
0.92
Gossypium hirsutum (Malvaceae)
1.0
Citrus sinensis (Rutaceae)
Manihot esculenta (Euphorbiaceae)
0.99
Populus trichocarpa (Salicaceae)
0.99
0.95
Quercus suber (Fagaceae)
Morus nota (Moraceae)
0.95
Glycine max (Leguminoseae)
1.0
Cucurbis maxima (Cucurbitaceae)
Arabidopsis thaliana (Brassicaceae)
0.97
Raphanus sativus (Brassicaceae)
1.0
Brassica rapus (Brassicaceae)
0.99
Physcomitrella patens (Funariaceae, bryophyta)
Ceratodon purpureas (Ditrichaceae, bryophyta)
1.0
