# Supplementary material for: CCDC22 and CCDC93, two potential retriever-interacting proteins, are required for root and root hair growth in Arabidopsis
Source: Front Plant Sci. 2022 Dec 22;13:1051503. doi: 10.3389/fpls.2022.1051503 (PMC9815543; doi:10.3389/fpls.2022.1051503)
Supplement: Supplementary Figure 6 — CCDC22 and CCDC93 expression levels in WT and their respective mutant backgrounds. RNA was extracted from 7-day old seedlings grown on 1X MS media. First strand cDNA was generated and used as template for qRT/PCR. Primers ccdc22_qRT1_F and ccdc22qRT1_R were used to quantitate level of CCDC22 transcript and primers ccdc93_qRT1_F and ccdc93qRT1_R were used to quantitate the level of CCDC93 transcript in wild type and mutant backgrounds. Expression levels were normalized to WT levels of ACT2 expression and compared using a student’s t-test. *p value < 0.05; *** p value <0.001. [file Presentation_6.pptx]

## Slide 1
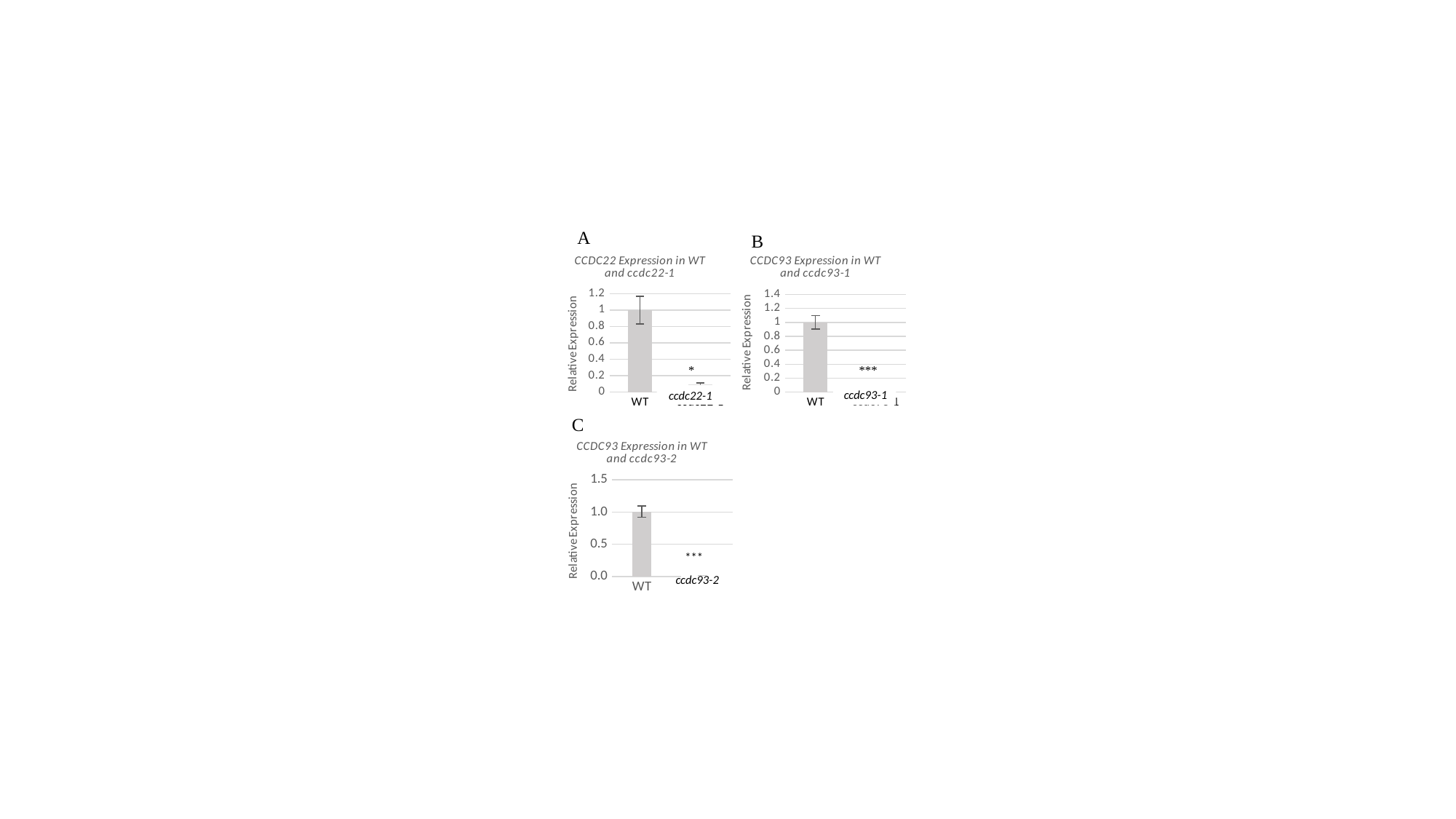

A
B
### Chart: CCDC22 Expression in WT and ccdc22-1
| Category | |
|---|---|
| WT | 1.0 |
| ccdc22-1 | 0.09177072154486428 |
### Chart: CCDC93 Expression in WT and ccdc93-1
| Category | |
|---|---|
| WT | 1.0 |
| ccdc93-1 | 0.00019944311983955806 |*
***
ccdc93-1
ccdc22-1
C
### Chart: CCDC93 Expression in WT and ccdc93-2
| Category | |
|---|---|
| WT | 1.0079377802797358 |
| 93-2 | 0.01168298856389479 |***
ccdc93-2
