# Supplementary material for: CCDC22 and CCDC93, two potential retriever-interacting proteins, are required for root and root hair growth in Arabidopsis
Source: Front Plant Sci. 2022 Dec 22;13:1051503. doi: 10.3389/fpls.2022.1051503 (PMC9815543; doi:10.3389/fpls.2022.1051503)
Supplement: Supplementary Figure 7 — Genotyping of ccdc22-1 and the CCDC22:CCDC22-RFP (ccdc22-1) transgenic line. (A) Visualization of the amplification of PCR products from isolated genomic DNA in which the following primer combinations were used: Primer 1) ccdc22_pENTR_F/ccdc22_R_STOP 2) LB1.3/ccdc22_pENTR_F 3) 22proSacIpENTR_F/RFP_R. See Table S1 for primer sequences. (B) Gene model for the transgenic RFP fusion construct with the locations of the indicated primer pairs. Image is drawn to scale. [file Presentation_7.pptx]

## Slide 1
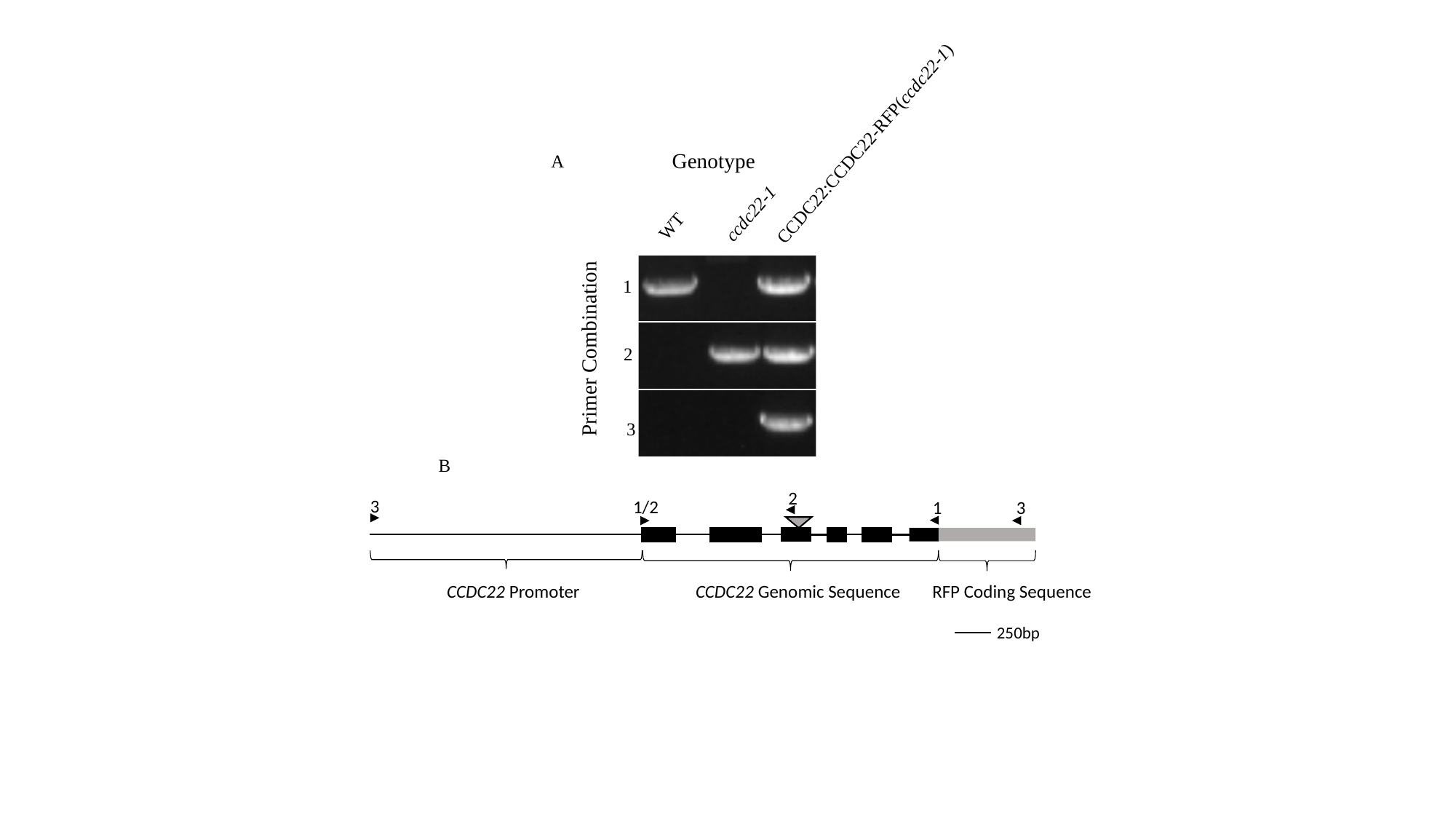

CCDC22:CCDC22-RFP(ccdc22-1)
Genotype
ccdc22-1
WT
1
Primer Combination
2
3
A
B
2
3
1/2
1
3
CCDC22 Promoter
RFP Coding Sequence
CCDC22 Genomic Sequence
250bp
