# Supplementary material for: CCDC22 and CCDC93, two potential retriever-interacting proteins, are required for root and root hair growth in Arabidopsis
Source: Front Plant Sci. 2022 Dec 22;13:1051503. doi: 10.3389/fpls.2022.1051503 (PMC9815543; doi:10.3389/fpls.2022.1051503)
Supplement: Supplementary Figure 8 — Genotyping of ccdc93-1 and the CCDC93:CCDC93-RFP (ccdc93-1) transgenic line. (A) Visualization of the amplification of PCR products from isolated genomic DNA in which the following primer combinations were used: 1) ccdc93_pENTR_F/ccdc93_R_STOP; 2) ccdc93_pENTR_F/LB1.3; 3) 93proSacIpENTR_F/RFP_R. See Table S1 for the primer sequences. (B) Gene model for the transgenic RFP fusion construct with the locations of the indicated primer pairs. Image is drawn to scale. [file Presentation_8.pptx]

## Slide 1
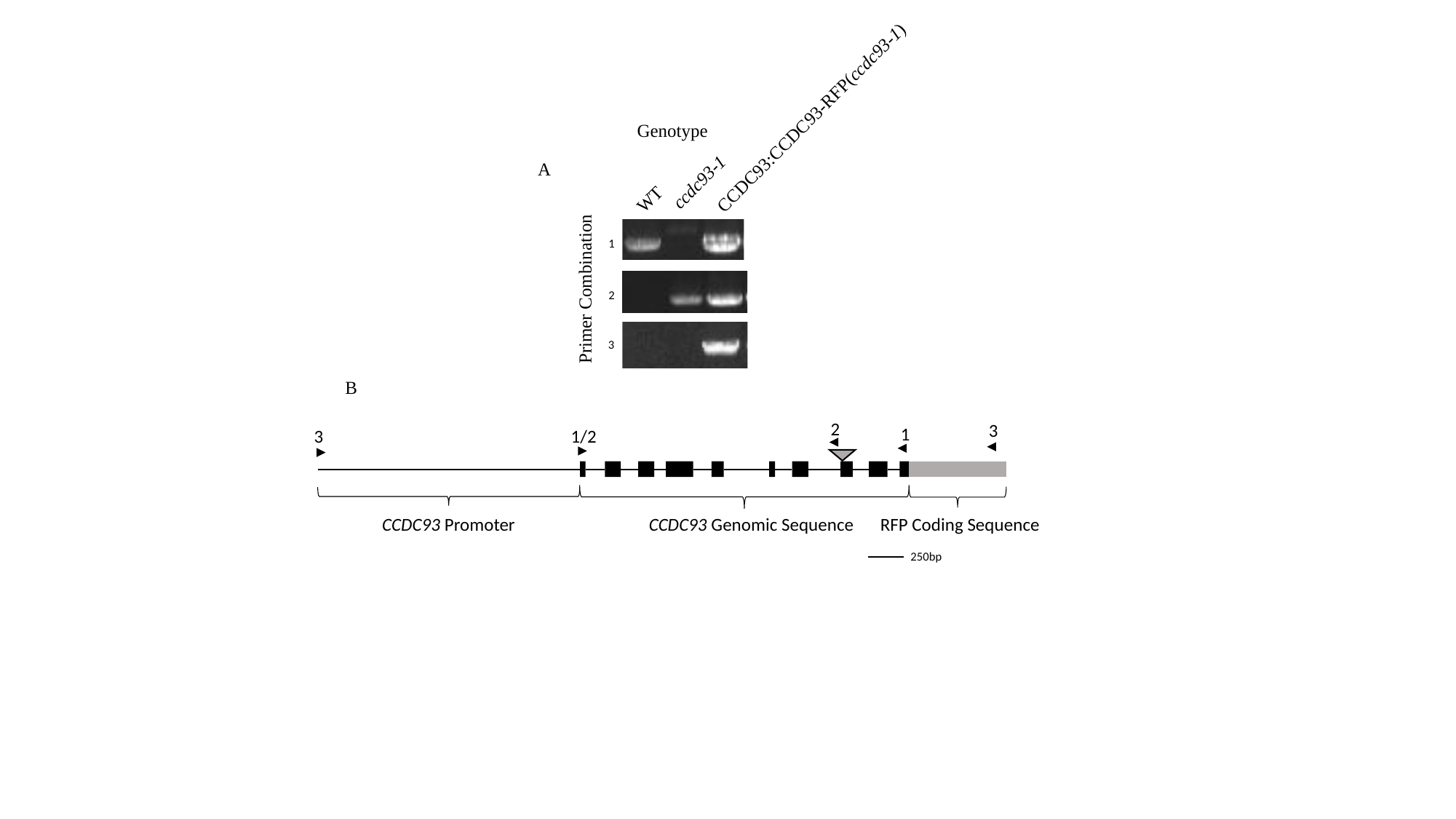

CCDC93:CCDC93-RFP(ccdc93-1)
Genotype
ccdc93-1
WT
1
Primer Combination
2
3
A
B
2
3
1
1/2
3
CCDC93 Promoter
RFP Coding Sequence
CCDC93 Genomic Sequence
250bp
