# Supplementary material for: CCDC22 and CCDC93, two potential retriever-interacting proteins, are required for root and root hair growth in Arabidopsis
Source: Front Plant Sci. 2022 Dec 22;13:1051503. doi: 10.3389/fpls.2022.1051503 (PMC9815543; doi:10.3389/fpls.2022.1051503)
Supplement: Supplementary file 11 [file DataSheet_1.pdf]

Name	Sequence (5' – 3')	Purpose
ccdc22_pENTR_F	CACCATGGAGGAGGAATCGCGAG	Cloning *CCDC22* into pENTR/D-TOPO, Genotyping, cDNA amplification
ccdc22_R_STOP	TTATTTCTTAAGGCTTTCGTTTTCTTTCC	Cloning *CCDC22* into pENTR/D-TOPO, Genotyping, cDNA amplification
ccdc22_R_nSTOP	TTTCTTAAGGCTTTCGTTTTCTTTCC	Cloning *CCDC22* into pENTR/D-Topo
ccdc93_pENTR_F	CACCATGGAGAAAGACGATAACAGTGAC	Cloning *CCDC93* into pENTR/D-TOPO, Genotyping, cDNA amplification
ccdc93_R_STOP	TCAGAGGTGTTCGTTCATGGC	Cloning *CCDC93* into pENTR/D-TOPO, Genotyping, cDNA amplification
ccdc93_R_noSTOP	GAGGTGTTCGTTCATGGCAGA	Cloning *CCDC93* into pENTR/D-TOPO
vti13_dtopo_F	CACCATGGAATATGGAAGTTTCACCGT	Genotyping
vti13_dtopo_R	TCACCTGGTGAGTTTGAAGT	Genotyping
LB1.3	ATTTTGCCGATTTCGGAAC	Genotyping T-DNA insertion mutants
22proSacIpENTR_F	CACCAGCTCCAGGAGGATATCTTCTAGATAATTCC	Cloning of *CCDC22* promoter into pENTR/D-TOPO, Genotyping
22proSpeIpEntr_R	ACTAGTCTTTTCTTCTCAAAACTTTCTGCTTCTCTTCAAAG	Cloning of *CCDC22* promoter into pENTR/D-TOPO
93proSacIpENTR_F	CACCGAGCTCGCTTGCTAGTGCTGG	Cloning of *CCDC93* promoter into pENTR/D-TOPO, Genotyping
93proSpeIpENTR_R	GTGACTAGTCACCAATTACCACGATTTCAGATACGAC	Cloning of *CCDC93* promoter into pENTR/D-TOPO
RFP_R	CTTGGCCATGTAGGTGGTCT	Genotyping of transgenic plants
22proMut_F	GAATCAAGGAACGAGTGTTCCATTGTG	Q5 site directed mutagenesis of *CCDC22* promoter
22promutR	ACACTTTTAGACTCTAACTAGGCCTTAGTTTAC	Q5 site directed mutagenesis of *CCDC22* promoter
93proMut_F	GTAATCTTTACGAGTCCTTTTGGCTCAGC	Q5 site directed mutagenesis of *CCDC93* promoter
93proMut_R	AACAAGAGTTGTTCACAATTTCTCAAGATCC	Q5 site directed mutagenesis of *CCDC93* promoter
CCDC22_qRT1_F	CCGATCATATGCTGTTGTGG	*CCDC22* quantitative gene expression analysis
CCDC22_qRT1_R	AAACTCATGCCTCGAGCGT	*CCDC22* quantitative gene expression analysis
CCDC93_qRT1_F	AGATTGACGATGTGCCATGC	*CCDC93* quantitative gene expression analysis

CCDC93_qRT1_R	CAATAAGCTTCACACGGCCA	*CCDC93* quantitative gene expression analysis
ACT2_qRT1_F	CTTGCACCAAGCAGCATGAA	Quantitative gene expression
ACT2_qRT1_R	CCGATCCAGACACTGTACTTCCTT	Quantitative gene expression
